# Qitu qushi formula ameliorates diabetic kidney disease potentially through gut microbiota-derived indole-3-propionic Acid–Mediated regulation of the Sirt1/FoxO1 pathway

**DOI:** 10.3389/fphar.2026.1802567

**Published:** 2026-06-02

**Authors:** Wenli Liu, Junhui Chen, Wenhua Gu, Yaohua Shen, Meifang Liu, Shiqi Wang, Zhaoyu Lu, Wei Mao, Xusheng Liu, Ruobing Wang, Lei Zhang

**Affiliations:** 1 Department of Nephrology, State Key Laboratory of Traditional Chinese Medicine Syndrome (The Second Clinical College of Guangzhou University of Chinese Medicine, The Second Affiliated Hospital of Guangzhou University of Chinese Medicine), Guangzhou, China; 2 Pok Oi Hospital WFAS Acupuncture Specialist Centre, Hong Kong, Hong Kong SAR, China

**Keywords:** diabetic kidney disease, gut microbiota, indole-3-propionic acid, qitu qushi formula, SIRT1/FOXO1, tryptophan metabolism

## Abstract

**Background:**

Diabetic kidney disease (DKD) is a leading cause of end-stage renal disease, and current pharmacotherapies provide limited renal protection. Qitu Qushi Formula (QTQSF), a traditional Chinese medicine prescription, has shown therapeutic potential in DKD, but its underlying mechanisms remain unclear. This study aimed to elucidate whether QTQSF alleviates DKD *via* gut microbiota-mediated pathways.

**Methods:**

Clinical, animal, and cellular studies were integrated to investigate the therapeutic effects of QTQSF. Clinical samples from 30 patients with type 2 DKD received QTQSF treatment for 6 months was used to assess renal function and gut microbiota. To explore microbiota-mediated mechanisms, db/db mice, pseudo-germ-free models, and fecal microbiota transplantation (FMT) were utilized. Multi-omics analyses, including 16S rRNA sequencing, untargeted and targeted metabolomics, and transcriptomics, were conducted to uncover key pathways underlying QTQSF’s efficacy.

**Results:**

QTQSF significantly improved renal function and remodeled gut microbial composition in DKD patients. In db/db mice, QTQSF reduced albuminuria, fibrosis, and apoptosis, whereas these protective effects were attenuated after gut microbiota depletion. FMT supported that gut microbiota mediated the renoprotective effects of QTQSF. Integrated multi-omics analyses revealed that QTQSF enhanced microbial tryptophan metabolism and increased the gut microbiota-derived metabolite indole-3-propionic acid (IPA). Elevated IPA levels were associated with regulation of the renal Sirt1/FoxO1 pathway, which was further validated in podocytes as a key mechanism underlying the anti-apoptotic effects.

**Conclusion:**

QTQSF may ameliorate DKD by enhancing gut microbiota-derived IPA production and regulating the Sirt1/FoxO1 signaling pathway, thereby attenuating renal injury. These findings provide mechanistic insight into the renoprotective effects of QTQSF and highlight a gut microbiota–metabolite–host signaling axis in DKD.

## Introduction

Diabetic kidney disease (DKD) is the most common and severe microvascular complication of diabetes, affecting up to 40% of diabetic patients and representing the leading cause of end-stage renal disease (ESRD) worldwide (Alicic et al., 2017). It is characterized by persistent proteinuria, progressive decline in estimated glomerular filtration rate (eGFR), and irreversible renal injury (Xie et al., 2024; Yong et al., 2025). Despite advances such as sodium-glucose cotransporter 2 (SGLT2) inhibitors and glucagon-like peptide-1 (GLP-1) receptor agonists, current treatments mainly targeting glycemic and blood pressure control remain insufficient to halt disease progression (Heerspink et al., 2018; Gerstein et al., 2019). Therefore, novel therapeutic strategies that provide additional renal protection are urgently needed.

Gut dysbiosis is considered an important factor in the pathogenesis of DKD, with patients exhibiting diminished gut microbiota diversity, characterized by a reduction in beneficial bacteria and an increase in pathogenic Enterobacteriaceae. Gut-derived metabolites, particularly those from the tryptophan pathway, are essential for renal health (Lin et al., 2025). Indole-3-propionic acid (IPA), a metabolite from the tryptophan indole pathway, has been shown to exert significant renal protective effects. Research indicated that IPA preserves renal function by stabilizing Sirtuin 1 (Sirt1), facilitating the deacetylation of PGC-1α in glomerular endothelial cells, and promoting mitochondrial biogenesis (Zeng et al., 2025). Sirt1, an NAD^+^-dependent deacetylase, activates FoxO transcription factors, thereby upregulating genes involved in antioxidant defenses and autophagy, which enhances mitochondrial function and stress resistance. Furthermore, activation of the Sirt1/FoxO1 axis has been reported to enhance autophagy, reduce reactive oxygen species, and mitigate inflammation, thereby alleviating proteinuria, glomerular fibrosis, and mitochondrial dysfunction (Wakino et al., 2015). However, evidence supporting the use of traditional Chinese medicine (TCM) to modulate gut microbiota and increase IPA levels for the treatment of DKD remains limited.

The Qitu Qushi Formula (QTQSF) is a patented formulation (Patent No. ZL202110551520.1) derived from the classical Chinese prescription Pingwei San and refined by clinical experts based on clinical experience. It is currently used as an empirical prescription at Guangdong Provincial Hospital of Chinese Medicine. Clinical evidence suggested that QTQSF slows renal function decline and reduces proteinuria in patients with stage 2–4 DKD, while maintaining a favorable safety profile (Liu M. et al., 2022; Liu M. et al., 2024). However, the underlying mechanism, particularly whether QTQSF confers renoprotection through modulation of the gut microbiota-metabolite-kidney axis, remains to be elucidated.

Given the pivotal role of the gut-kidney axis in DKD and the multi-target characteristics of TCM, we hypothesized that QTQSF may exert therapeutic effects by modulating gut microbiota, promoting beneficial metabolites, and activating renal protective pathways. This study integrated clinical, animal, and cellular experiments with multi-omics analyses to systematically elucidate the mechanisms underlying QTQSF’s therapeutic effects.

## Method and materials

### Experimental drugs

The therapeutic agent used in this study was QTQSF granules, with dapagliflozin (DAPA) serving as the positive control. QTQSF comprises seven botanical drugs: *Astragalus mongholicus Bunge [Fabaceae; Astragali mongholici radix], Centella asiatica (L.) Urb [Apiaceae; Centellae asiaticae herba], Cuscuta australis R. Br [Convolvulaceae; Cuscutae semen], Atractylodes lancea (Thunb.) DC [Asteraceae; Atractylodis lanceae rhizoma], Prunus davidiana (Carrière) Franch [Rosaceae; Persicae semen], Citrus × aurantium L [Rutaceae; Aurantii amari epicarpium et mesocarpium], Isaria cicadae Miquel [Cordycipitaceae; Isaria cicadae Miquel]*, with respective dosages of 20:15:15:10:10:10:8 g, according to the Chinese Pharmacopeia (2020 Edition) and clinical practice. The QTQSF granules were produced by Jiangyin Tianjiang Pharmaceutical Co., Ltd (Jiangsu, China) under Good Manufacturing Practice standards. Each sachet contained 7.5 g of concentrated extract granules. The clinical dosage was two sachets (15 g) per administration, administered twice daily, and dissolved in sterile water immediately before use. DAPA (Drug Approval No. J20170040) was obtained from the Pharmacy of Guangdong Provincial Hospital of Chinese Medicine (Guangdong, China). Tablets (10 mg) were finely ground and freshly dissolved in sterile water prior to administration.

### Chemical profiling of QTQSF granules

In accordance with the ConPhyMP guidelines (Heinrich et al., 2022), the plant metabolites in QTQSF granules, a multi-botanical drug formulation, were characterized using ultra-high-performance liquid chromatography coupled with tandem mass spectrometry (UHPLC–MS/MS). UHPLC–HRMS analysis was conducted at Shanghai Fuda Testing Technology Group Co., Ltd (Shanghai, China). Chromatographic separation was performed on an ACQUITY UPLC HSS T3 column (2.1 × 100 mm, 1.8 μm; Waters, Milford, MA, United States) at 35 °C with a flow rate of 0.3 mL/min, using 0.1% formic acid in water (A) and acetonitrile (B) as the mobile phase under gradient elution. Mass spectrometric analysis was conducted on a Q Exactive Plus Orbitrap system (Thermo Fisher Scientific, Bremen, Germany) equipped with an electrospray ionization (ESI) source operating in both positive and negative modes. Data were acquired in Full MS–ddMS^2^ mode over an m/z range of 100–1200, with resolutions of 70,000 (MS^1^) and 17,500 (MS^2^). Raw data were processed using Compound Discoverer software (version 3.2, Thermo Fisher Scientific, United States), and metabolite identification was achieved by matching against the mzCloud database (https://www.mzcloud.org/) and a local mzVault spectral library (Thermo Fisher Scientific), with a mass tolerance <5 ppm and a match score >70.

**FIGURE 1:**
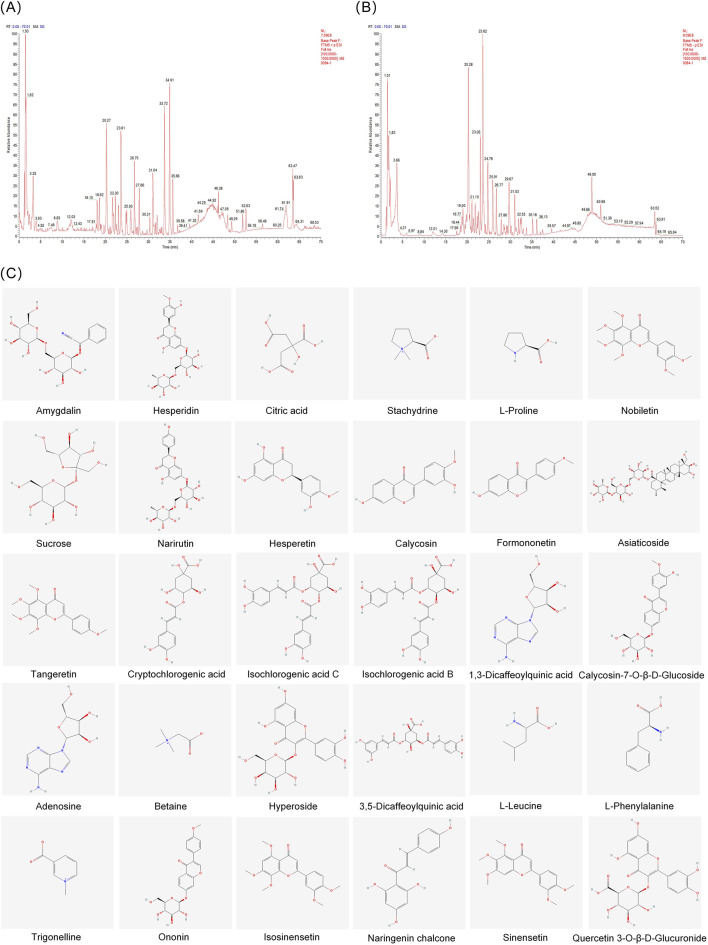
The base peak chromatogram of Qitu Qushi Formula granules in **(A)** negative ion mode and **(B)** positive ion mode. **(C)** Chemical structures of the top 30 metabolites ranked by relative abundance. Structures were obtained from the PubChem database (https://pubchem.ncbi.nlm.nih.gov/). Abbreviations: DKD, diabetic kidney disease; FMT, fecal microbiota transplantation; QTQSF, Qitu Qushi Formula.

The extracted ion chromatograms obtained in both positive and negative ionization modes are presented in Figures 1A,B. Major metabolites were relatively quantified based on peak areas, and the results are summarized in Supplementary Table S1. The chemical structures of the top 30 metabolites, ranked by relative abundance, were retrieved from the PubChem database (https://pubchem.ncbi.nlm.nih.gov/) and are shown in Figure 1C. Among the most abundant metabolites identified, representative bioactive metabolites included *glycosides and organic acids* (e.g., *amygdalin* and *citric acid*), *flavonoids* (*hesperidin, nobiletin, narirutin, hesperetin, tangeretin,* and *quercetin derivatives*), *isoflavones* (*calycosin, formononetin and their glycosides*), *triterpenoids* (*asiaticoside* and its minor *aglycone, asiatic acid*), *alkaloids* (*stachydrine*), and *phenolic acids* (*chlorogenic acid derivatives* such as *cryptochlorogenic acid* and *dicaffeoylquinic acids*). These metabolites, identified as relatively abundant constituents of the QTQSF botanical drug formulation, have been widely reported to exhibit anti-inflammatory, metabolic regulatory, renal protection and microbiota-modulating activities, which may provide a phytochemical basis for the multi-target pharmacological effects of QTQSF.

### Clinical samples

Serum and fecal samples from patients with type 2 DKD were obtained from a previously published single-arm clinical study of QTQSF conducted at the Guangdong Provincial Hospital of Chinese Medicine (Guangdong, China) between May 2020 and December 2021 (Ethics No. BF2020-067-01) (Liu M. et al., 2022). Eligible cases had completed clinical and follow-up data, completed the 6-month QTQSF treatment, demonstrated good adherence (≥80% of prescribed doses), and had complete follow-up data available for analysis. During the study period, all patients received routine treatment according to disease stage, in accordance with current clinical guidelines for DKD and CKD stages 3–5, as well as integrated Chinese and Western medicine management protocols. Patients with incomplete records, poor compliance, or treatment discontinuation were excluded. Fecal and serum samples were collected at baseline and after 6 months of treatment. Informed consent was obtained from all subjects prior to participation.

### *In Vivo* studies

#### Experimental animals

Five-week-old male db/db mice (n = 40) and db/m mice (n = 5) were purchased from Changzhou Cavens Laboratory Animal Co., Ltd (Changzhou, China; License No. SCXK(Su)2021–0013). All animals were maintained in a specific pathogen-free (SPF) facility at Guangzhou University of Chinese Medicine (Guangzhou, China; License No. SYXK(Yue)2023–0347) under controlled conditions (temperature, 22 °C ± 2 °C; humidity, 50% ± 5%; 12h light/dark cycle), with free access to standard chow and water. After a 7-day acclimatization period, the experimental procedures were initiated. All protocols were approved by the Animal Ethics Committee of Guangzhou University of Chinese Medicine (Approval No. 20240322003).

### Establishment of the pseudo-germ-free (PGF) model

A PGF model was developed by administering antibiotics (ABX) containing ampicillin (1 g/L), metronidazole (1 g/L), vancomycin (0.5 g/L), and neomycin (1 g/L) (Macklin, Shanghai, China) in sterile drinking water for 2 weeks to deplete the intestinal microbiota (Xian et al., 2022). Model establishment was validated through quantitative fecal DNA analysis, 16S rRNA gene sequencing, and α-diversity assessment. Successful microbiota depletion was confirmed when all P values were <0.05. In both the QTQSF intervention and fecal microbiota transplantation (FMT) experiments, antibiotics treatment significantly reduced fecal bacterial DNA levels (P < 0.05) and markedly decreased α-diversity indices (P < 0.001) (Supplementary Table S2; Supplementary Figure S1). At the phylum level, untreated mice exhibited gut microbiota dominated by Bacteroidota and Firmicutes, whereas these taxa were substantially diminished following antibiotic exposure, with Proteobacteria becoming predominant (Figures 1C,D). Collectively, these results confirmed the successful establishment of the PGF model.

### QTQSF intervention

Following 1 week of acclimatization, db/db mice (n = 25) were randomly assigned to five groups (n = 5 per group): Model, DAPA, L-QTQSF, H-QTQSF, and ABX-H-QTQSF. Db/m mice constituted the Normal group. Mice in the ABX-H-QTQSF group underwent PGF modeling for 2 weeks, while the other groups were maintained under standard conditions. Post-modeling, the respective interventions were administered *via* oral gavage once daily for 12 weeks as follows: the DAPA group received dapagliflozin (0.15 mg/mL), the L-QTQSF group received QTQSF at 227.5 mg/mL, and both the H-QTQSF and ABX-H-QTQSF groups received QTQSF at 455 mg/mL. The Model and Normal groups received an equivalent volume of distilled water. The gavage volume was standardized at 0.1 mL per 10 g of body weight.

## FMT

Following 1 week of acclimatization, fifteen db/db mice were rendered PGF using the previously described antibiotics regimen and subsequently divided into three groups (n = 5 per group): ABX, H-QTQSF + FMT, and ABX-H-QTQSF + FMT. For fecal suspension preparation, fresh fecal samples were collected from the H-QTQSF and ABX-H-QTQSF donor groups after 8 weeks of QTQSF intervention. The feces were suspended in sterile saline (1:5, w/v), homogenized for 10 min, vortexed for 1 min, and stirred at room temperature for 10 min. The homogenate was then filtered through a 100 μm cell strainer, aliquoted into sterile tubes, supplemented with 20% (v/v) glycerol, and stored at −80 °C until use. Recipient mice in the H-QTQSF + FMT and ABX-H-QTQSF + FMT groups received fecal suspensions from the H-QTQSF and ABX-H-QTQSF donor groups, respectively, while the ABX group received an equivalent volume of sterile saline. FMT was administered by oral gavage (0.2 mL per mouse) once daily for 10 weeks.

### Sample collection and biochemical measurements

During the experimental period, body weight was recorded weekly, fasting blood glucose (FBG) was measured biweekly using a glucometer (Bayer, Bergkamen, Germany), and urinary albumin-to-creatinine ratio (UACR) was assessed every 4 weeks. At the experimental endpoint, mice were fasted for 8 h and then euthanized. Blood, kidney tissues, and fecal samples were collected. Each kidney was divided into two portions: one fixed in 4% paraformaldehyde for histological analysis, and the other stored at −80 °C for molecular assays. Fecal samples were also preserved at −80 °C. UACR, serum creatinine (SCr) and glycated hemoglobin (HbA1c) levels were determined using a Cobas C702 automatic biochemical analyzer (Roche, Mannheim, Germany). Serum and renal indole-3-propionic acid (IPA) concentrations were quantified using an enzyme-linked immunosorbent assay kit (ZC-53467-J, ZCIBIO, China) following the manufacturer’s instructions.

### Histopathological analysis

Histopathological examination of kidney tissues was performed using standard procedures. Fixed tissues were dehydrated through graded ethanol, cleared in xylene, and embedded in paraffin. Sections of 3 μm thickness were prepared and stained with hematoxylin-eosin (H&E; AR1180-100, Boster, China), periodic acid-Schiff (PAS; DG0005, Leagene, China), and Masson’s trichrome (DC0033, Biochannel, China). The extent of renal fibrosis was quantitatively evaluated using ImageJ (version 1.51j8, National Institutes of Health, Bethesda, MD, United States). For each sample, five randomly selected fields were analyzed, and the fibrotic area was expressed as the percentage of collagen-positive area.

### Western blot analysis

Kidney tissues were lysed in RIPA buffer supplemented with protease and phosphatase inhibitors. After ultrasonic disruption, lysates were centrifuged at 20,817 × g for 15 min at 4 °C, and the supernatant was collected. Protein concentrations were determined using a bicinchoninic acid assay. Equal amounts of protein were separated by SDS-PAGE and transferred onto polyvinylidene difluoride membranes. Primary antibodies included α-sma (#19245, CST, United States), Vimentin (sc-66002, Santa Cruz, United States), Bax (#14796, CST, United States), Bcl-2 (ab182858, Abcam, United States), total caspase-3 (Cas-3) (#9662, CST, United States), cleaved caspase-3 (C-Cas-3) (#9664, CST, United States), Sirt1 (#8469, CST, United States), FoxO1 (sc-374427, Santa Cruz, United States), acetylated FoxO1 (ac-FoxO1) (AF2305, Affinity, Suzhou, China), Fas-L (sc-19681, Santa Cruz, United States), Synaptopodin (sc-515842, Santa Cruz, United States), Nephrin (ab216341, Abcam, United States), and GAPDH (#5174, CST, United States). HRP-conjugated secondary antibodies were anti-mouse IgG (#7076, CST, United States) and anti-rabbit IgG (#7074, CST, United States). Protein bands were visualized using enhanced chemiluminescence, and band intensities were quantified using ImageJ software (version 1.51j8, National Institutes of Health, Bethesda, MD, United States) with normalization to GAPDH.

### *In Vitro* study

The mouse podocyte cell line MPC-5 was cultured in RPMI-1640 medium (11875093, Gibco, United States) supplemented with 10% fetal bovine serum (0218072801–1, MP Biomedicals, United States) at 37 °C in a humidified atmosphere containing 5% CO_2_. Cells were divided into six groups: Normal, Model, L-IPA, H-IPA, L-IPA + EX-527, and H-IPA + EX-527. After 24 h of seeding, cells in the Model group were exposed to 30 mM glucose for 24 h. The L-IPA group was treated with 30 mM glucose and 25 μM IPA (HY-W015229, MCE, United States), and the H-IPA group with 30 mM glucose and 50 μM IPA for 24 h. The L-IPA + EX527 and H-IPA + EX527 groups were co-treated with 10 μM EX-527 (Sirt1 inhibitor; HY-15452, MCE, United States) in addition to 30 mM glucose and 25 or 50 μM IPA, respectively. Cell viability was determined using a Cell Counting Kit-8 (CCK-8; CK04-500, Dojindo, Japan), with optical density measured at 450 nm in six independent replicates per sample. Western blotting was performed as previously described.

### Gut microbiota 16S rRNA gene sequencing

Fecal DNA was extracted using the E. Z.N.A.® Soil DNA Kit (Omega Bio-tek, United States), and DNA quality was assessed with a NanoDrop 2000 spectrophotometer (Thermo Fisher, United States). The V3-V4 regions of the bacterial 16S rRNA gene were amplified using barcoded primers 338F (5'-ACT​CCT​ACG​GGA​GGC​AGC​AG-3') and 806R (5'-GGACTACHVGGGTWTCTAAT-3'), purified with Agencourt AMPure XP beads (Beckman Coulter, United States), and quantified with a Qubit 4.0 fluorometer (Thermo Fisher, United States). Sequencing was performed on the Illumina NovaSeq 2000 platform (MJBIO, China) with 2 × 150 bp paired-end reads, generating at least 30,000 reads per sample (Ren et al., 2022). Raw reads were filtered using Fastp v0.19.6 and merged with FLASH v1.2.11. Operational taxonomic units (OTUs) were clustered at 97% similarity using UPARSE v7.1, and taxonomy was assigned *via* the RDP Classifier v2.11 against the Silva 138 database. Alpha and beta diversity were analyzed using Mothur v1.30.2 and QIIME v1.9.1 (Bray-Curtis distance, PCoA). Community structure differences were visualized by partial least squares discriminant analysis (PLS-DA), and differential taxa were identified using linear discriminant analysis effect size (LEfSe) (LDA >1, P < 0.05).

### Untargeted metabolomic analysis

Untargeted metabolomics was performed on fecal samples to characterize gut microbial metabolic profiles. Freeze-dried fecal samples (50 mg) were extracted with 400 μL of pre-chilled methanol/water (4:1, v/v) containing 0.02 mg/mL L-2-chlorophenylalanine as an internal standard. Samples were homogenized at −10 °C for 6 min using a cryogenic tissue grinder (Wonbio-96E, Wanbo, Shanghai, China), ultrasonicated at 5 °C (40 kHz, 30 min), incubated at −20 °C for 30 min, and centrifuged at 13,000×g for 15 min at 4 °C. Supernatants were filtered through 0.22 μm membranes, and quality control (QC) samples were injected every five runs to monitor system stability. Metabolomic profiling was conducted on a UHPLC–Q Exactive HF-X system (Thermo Fisher, United States) with an HSS T3 column (100 × 2.1 mm, 1.8 μm; Waters, United States) in both positive and negative ESI modes (m/z 70–1050). Data were processed in Progenesis QI v3.0 (Waters, United States) for peak alignment and metabolite annotation against HMDB 4.0, Metlin 2.0, and an in-house library (mass tolerance <5 ppm, isotope similarity >800, MS/MS score >60%). Features present in ≥80% of samples with QC RSD <30% were retained. The preprocessed data matrix was subjected to multivariate statistical analysis using the ropls package (version 1.6.2) in R. Principal component analysis (PCA) was first performed to visualize the overall distribution and clustering of samples. Orthogonal PLS-DA was subsequently conducted to identify metabolic differences between groups. Differential metabolites were defined by VIP>1 and P < 0.05 (Wilcoxon test), and KEGG pathway enrichment was performed using SciPy v1.0.0 (Fisher’s exact test).

### Targeted tryptophan metabolomic analysis

Targeted metabolomics focused on tryptophan pathway metabolites in serum to evaluate their systemic circulation. Serum samples (100 μL) were mixed with 10 μL of internal standard solution ([indole-2H5]-L-tryptophan, Trp-D5, 4000 ng/mL; catalog no. IR-22745, ZZStandard, Shanghai, China; purity 99%, isotopic enrichment 98 atom% D) and 390 μL of methanol, vortexed, and ultrasonicated at 5 °C (40 kHz) for 30 min. After incubation at −20 °C for 30 min, samples were centrifuged at 13,000×g (4 °C, 15 min). The supernatant (350 μL) was evaporated under nitrogen, reconstituted in 70 μL of 1% acetonitrile containing 0.1% formic acid, ultrasonicated at low temperature for 15 min, and centrifuged again for analysis. Chromatographic separation was performed on an AB SCIEX QTRAP 6500+ LC-MS/MS system (AB SCIEX, United States) equipped with a Waters ACQUITY UPLC HSS T3 column (2.1 × 150 mm, 1.8 μm) at 40 °C, with a flow rate of 0.35 mL/min and injection volume of 2 μL. Quantification of 33 tryptophan metabolites was achieved in both positive and negative MRM modes. Calibration curves with 11 concentration points were constructed using weighted linear regression, and metabolite concentrations were calculated from peak area ratios. QC samples were injected every batch in randomized order to monitor analytical stability. Data analysis was performed on the Majorbio Cloud 3.0 platform. PCA and OPLS-DA were used for multivariate analysis, and differential metabolites were defined by VIP >1 and P < 0.05.

### Renal transcriptomic sequencing

Total RNA was extracted from renal tissues using Qiazol (Qiagen, Germany) and purified with an RNA Purification Kit (MJBIO, China). RNA integrity was evaluated with a NanoDrop 2000 spectrophotometer (Thermo Fisher, United States; OD260/280 = 1.8–2.2) and an Agilent 5300 Bioanalyzer (Agilent, United States; RQN >6.5). Poly(A)+ mRNA was enriched using Oligo (dT) magnetic beads, fragmented, and reverse-transcribed to cDNA for library construction following the Illumina TruSeq protocol. Sequencing was performed on the Illumina NovaSeq X Plus platform with 150 bp paired-end reads, achieving ≥30 million reads per sample and Q30 ≥ 85%. Clean reads were aligned to the mouse reference genome (GRCm39) using STAR v2.7.1a. Differentially expressed genes were identified with DESeq2 (FDR <0.05, |log_2_FC| ≥1) and adjusted by the Benjamini–Hochberg method for multiple testing. KEGG pathway enrichment was analyzed by Fisher’s exact test (P < 0.05) and visualized using ClusterProfiler (R v4.2.2). All sequencing data passed FastQC quality control (Q30 > 80%, GC content 40%–60%) without detectable batch effects.

### Statistical analysis

Statistical analyses were conducted using GraphPad Prism 10. Data are presented as mean ± standard error of the mean (SEM). Differences between two groups were assessed using the Student’s t-test, while comparisons among multiple groups were performed using one-way analysis of variance (ANOVA). A two-tailed P value <0.05 was considered statistically significant.

## Results

### QTQSF improved renal function and modulated gut microbiota in DKD patients

**FIGURE 2:**
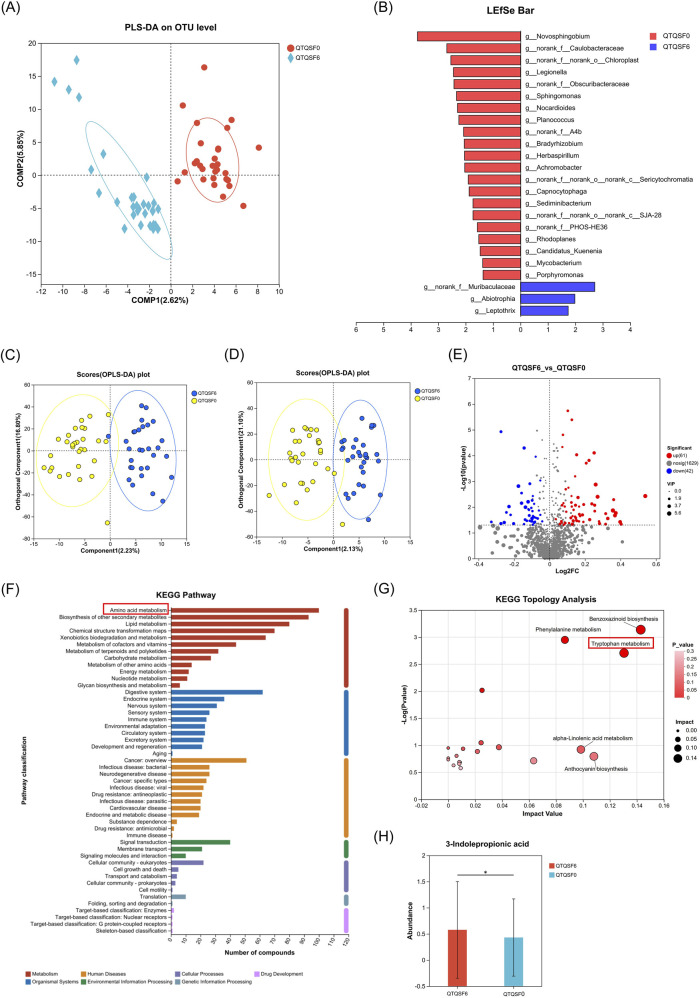
QTQSF modulated gut microbiota and tryptophan-indole metabolism in DKD patients. **(A)** PLS-DA score plot of fecal microbiota profiles between the QTQSF0 and QTQSF6 groups. **(B)** LEfSe-derived LDA score histogram showing differentially abundant genera between QTQSF0 and QTQSF6 groups. The length of each bar represents the effect size (LDA score). Taxonomic annotations (g__) denote genus-level classification. Differential taxa were identified based on LDA >1 and P < 0.05. **(C,D)** Untargeted fecal metabolomics: identified metabolites in positive- **(C)** and negative-ion mode. **(D) (E)** Volcano plot of differential fecal metabolites. **(F)** KEGG pathway annotation of differential metabolites. **(G)** KEGG pathway enrichment bubble plot based on topology analysis. **(H)** Targeted serum tryptophan metabolomics showing indole-3-propionic acid levels before and after QTQSF treatment. Abbreviations: QTQSF, Qitu Qushi Formula; DKD, diabetic kidney disease; PLS-DA, partial least squares discriminant analysis; LEfSe, linear discriminant analysis effect size; LDA, linear discriminant Analysis; KEGG, Kyoto Encyclopedia of Genes and Genomes; QTQSF0 and QTQSF6, before and after 6 months of QTQSF treatment, respectively.

A total of 30 eligible case samples from type 2 DKD patients were selected. These cases represented moderate disease severity with balanced baseline characteristics (age 64.3 ± 1.7 years; 56.7% male and 43.3% female; baseline SCr 139.5 μmol/L; eGFR 45.32 mL/min/1.73 m^2^; UACR 709.045 mg/g) (Table 1). After 6 months of QTQSF treatment, renal function improved significantly. SCr decreased from 139.5 (87.3–185.0) to 123.5 (50.3–178.0) μmol/L (P = 0.010), and eGFR increased from 45.32 (26.43–77.81) to 51.56 (30.83–82.94) mL/min/1.73 m^2^ (P = 0.050), while no improvement in UACR was observed (Table 1). Given these clinical improvements, we next investigated whether QTQSF modulated gut microbial composition. Although alpha-diversity indices (Chao, Shannon) showed no significant change (P = 0.388, 0.233) (Supplementary Figure S3), PLS-DA revealed clear microbial separation post-treatment (Figure 2A). LEfSe identified 24 differential genera, including 21 downregulated and three upregulated taxa (LDA>1,P < 0.05) (Figure 2B). These findings suggested that QTQSF improved renal function and modulated gut microbial composition in DKD patients, primarily reflecting compositional shifts.

**TABLE 1:** Comparison of clinical parameters between before and after treatment in patients with DKD.

Parameter	Before treatment, (IQR)	After treatment, (IQR)	P value
SCr (μmoL/L)	139.500 (87.250-185.000)	123.500 (50.300-178.000)	0.01
eGFR (ml/min/1.73m^2)^	45.320 (26.430-77.810)	51.560 (30.830-82.940)	0.05
UACR (mg/g)	709.045 (199.860-1649.120)	929.445 (347.563-2933.878)	0.001
HbA1c (%)	7.000 (6.300-7.475)	6.850 (6.175-8.000)	0.952

Abbreviations: DKD, diabetic kidney disease; SCr, serum creatinine; eGFR, estimated glomerular filtration rate; UACR, urinary albumin-to-creatinine ratio; HbA1c, glycated hemoglobin A1c; IQR, interquartile range.

### QTQSF activated tryptophan metabolism and elevated IPA levels in DKD patients

Untargeted fecal metabolomics of the 30 DKD patients, utilizing pre- and post-treatment samples, identified 6,741 features, with 103 differential metabolites (OPLS-DA, VIP>1, P < 0.05), including 61 upregulated and 42 downregulated metabolites (Figures 2C–E; Supplementary Table S3). Kyoto Encyclopedia of Genes and Genomes (KEGG) annotation of the differential metabolites provided a descriptive overview of their functional categories and showed that a substantial proportion were related to amino acid metabolism (Figure 2F). To further identify biologically relevant pathways associated with QTQSF treatment, KEGG pathway enrichment analysis was performed, revealing that tryptophan metabolism (P = 0.015, impact value = 0.130) was prominently affected, along with phenylalanine metabolism and benzoxazinoid biosynthesis (Figure 2G). Specifically, several tryptophan-related metabolites were altered, including increased indole-3-acetaldehyde and decreased three-indoleacetic acid and L-formylkynurenine, indicating a pathway-level alteration of microbial tryptophan metabolism within the gut environment (Supplementary Table S4). To determine whether these changes translated into systemic metabolic alterations, targeted tryptophan metabolomics was conducted using serum samples. Notably, among all measured tryptophan metabolites, indole-3-propionic acid (IPA, C_11_H_11_NO_2_; CAS No. 830–96–6) was the only metabolite significantly increased following QTQSF treatment (VIP = 2.57, P = 0.032) (Figure 2H; Supplementary Table S4). These results suggested that QTQSF may exert renoprotective effects in DKD patients by modulating gut microbiota and enhancing microbial tryptophan-indole metabolism, with IPA potentially serving as a key effector metabolite in gut microbiota–host interactions.

### QTQSF attenuated renal injury and apoptosis, and increased IPA levels in DKD mice

**FIGURE 3:**
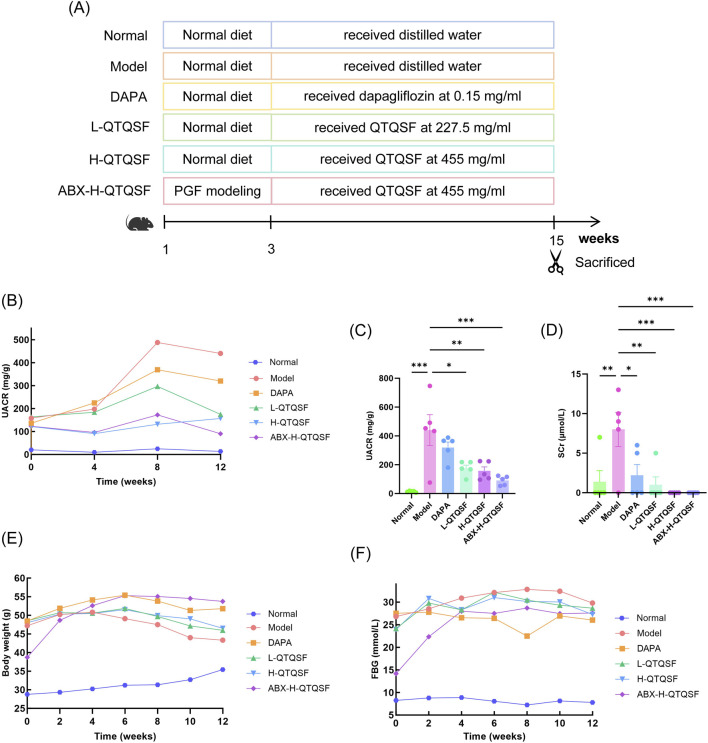
QTQSF improved renal function in DKD mice. **(A)** Study design and treatment schedule. Physiological parameters including UACR **(B,C)**, SCr **(D)**, body weight **(E)**, and FBG **(F)**. Data are presented as mean ± SEM (n = 5 per group). *P < 0.05, **P < 0.01, ***P < 0.001, ****P < 0.0001. Abbreviations: QTQSF, Qitu Qushi Formula; DKD, diabetic kidney disease; UACR, urinary albumin-to-creatinine ratio; SCr, serum creatinine; FBG, fasting blood glucose; SEM, standard error of the mean; ABX, antibiotics.

**FIGURE 4:**
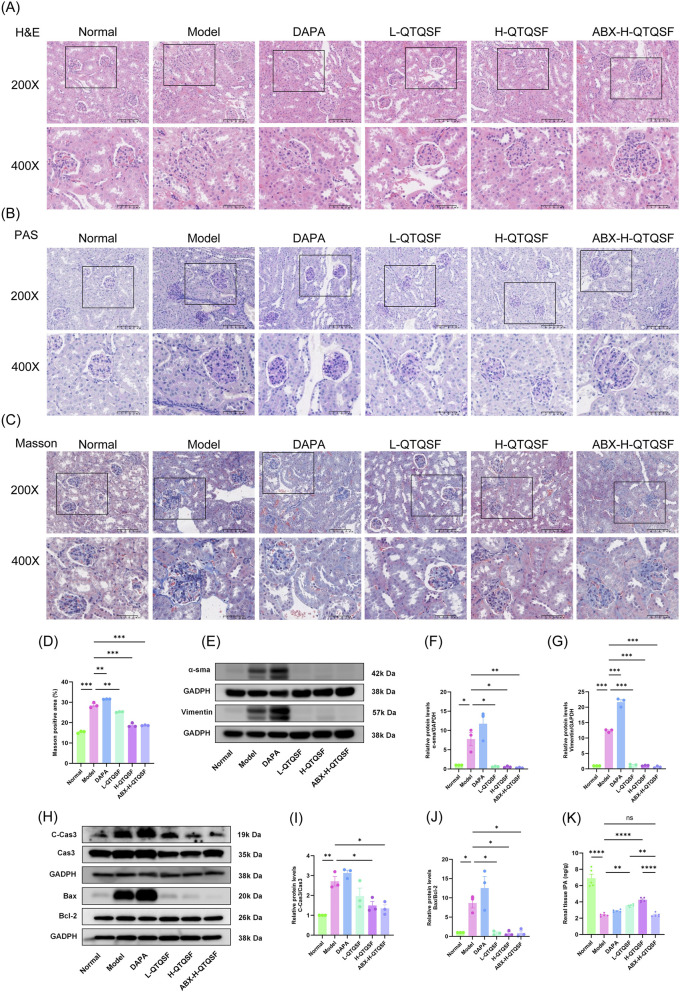
QTQSF attenuated renal injury and apoptosis, and increased renal IPA level in DKD mice. H&E **(A)**, PAS **(B)** and Masson staining **(C)**, with fibrosis quantification **(D)**. **(E–G)** Western blots and densitometry of α-sma and Vimentin. **(H–J)** Western blots and densitometry of C-Cas3/Cas3 and Bax/Bcl-2. **(K)** Renal IPA levels. Data are presented as mean ± SEM (n = 3 per group). *P < 0.05, **P < 0.01, ***P < 0.001, ****P < 0.0001. Abbreviations: QTQSF, Qitu Qushi Formula; DKD, diabetic kidney disease; H&E, hematoxylin and eosin; PAS, periodic acid–Schiff; C-Cas3/Cas3, cleaved caspase-3/total caspase-3; IPA, indole-3-propionic acid; SEM, standard error of the mean; ABX, antibiotics.

Using a spontaneous DKD mouse model, we evaluated the therapeutic effects of QTQSF and further explored the potential contribution of the gut microbiota to its efficacy (Figure 3A). After 12 weeks of treatment, UACR was markedly reduced in the L-QTQSF (P = 0.010), H-QTQSF (P = 0.006), and ABX-H-QTQSF (P < 0.001) groups compared with the Model group (Figures 3B,C). Similarly, SCr levels were significantly decreased in the L-QTQSF (P = 0.049), H-QTQSF (P = 0.009), and ABX-H-QTQSF (P = 0.009) groups (Figure 3D). No statistically significant differences were observed in body weight and FBG (Figures 3E,F). Compared with the Model group, histological analyses (H&E, PAS) showed improved glomerular architecture, reduced basement membrane thickening, and alleviated mesangial expansion in QTQSF-treated mice (Figures 4A,B). Besides, Masson staining confirmed reduced renal fibrosis across QTQSF-treated groups (P < 0.05) (Figures 4C,D). Western blotting demonstrated decreased α-sma, and Vimentin expression (Figures 4E–G), along with lower apoptotic markers including C-Cas3/Cas3 and Bax/Bcl-2 ratios (P < 0.05) (Figures 4H–J) after QTQSF treatment. Notably, renal tissue IPA levels increased significantly in the H-QTQSF group (P = 0.010) but not in the ABX-H-QTQSF group, highlighting the modulatory effect of QTQSF on gut microbiota and the crucial role of gut microbiota in IPA production (Figure 4K). Collectively, these findings suggested that QTQSF improves renal function, and attenuates fibrosis and apoptosis in DKD mice, with these effects potentially associated with changes in IPA levels.

### FMT explored the gut microbiota-mediated therapeutic effect of QTQSF in DKD mice

**FIGURE 5:**
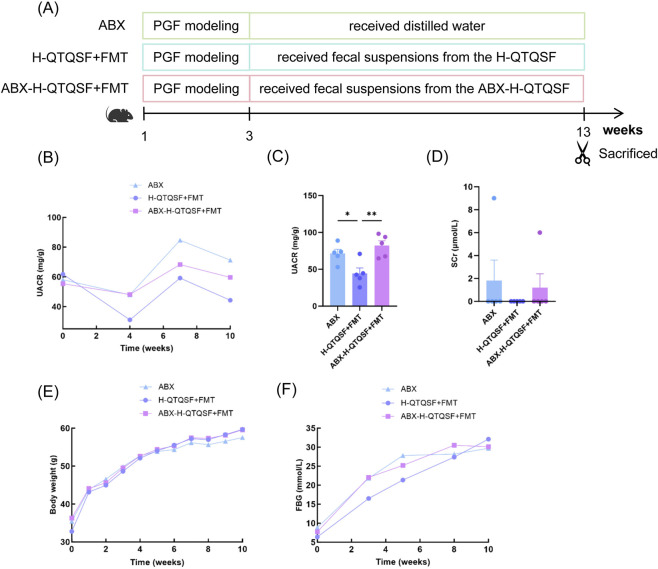
FMT from QTQSF-treated mice improved renal function in DKD mice. **(A)** Experimental design of the FMT study. PGF db/db recipient mice received fecal microbiota from H-QTQSF or ABX-H-QTQSF donor mice. Physiological parameters including UACR **(B,C)**, SCr **(D)**, body weight **(E)**, and FBG **(F)**. Data are presented as mean ± SEM (n = 5 per group). *P < 0.05, **P < 0.01, ***P < 0.001, ****P < 0.0001. Abbreviations: FMT, fecal microbiota transplantation; QTQSF, Qitu Qushi Formula; DKD, diabetic kidney disease; PGF, pseudo-germ-free; UACR, urinary albumin-to-creatinine ratio; SCr, serum creatinine; FBG, fasting blood glucose; SEM, standard error of the mean; ABX, antibiotics.

**FIGURE 6:**
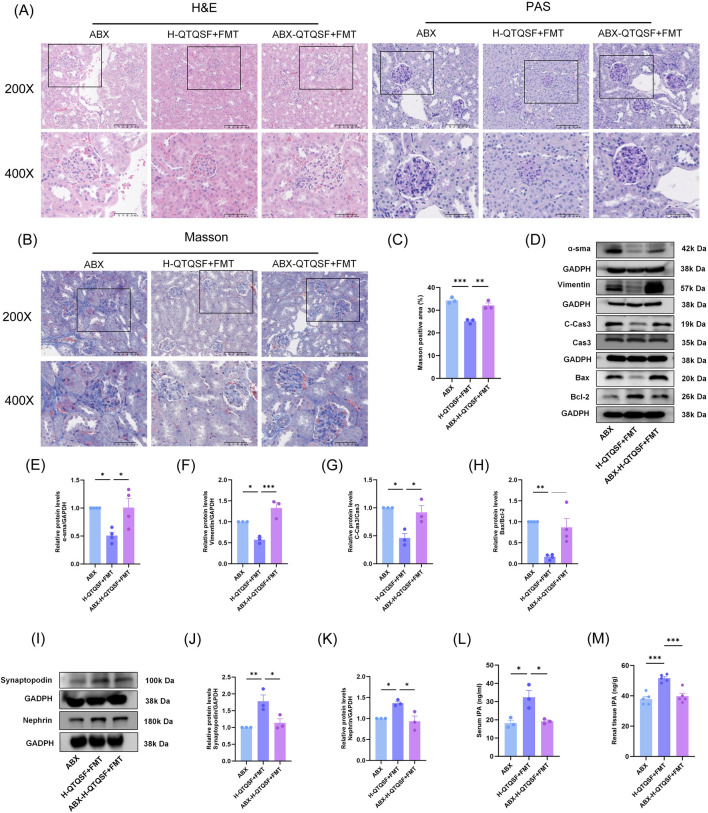
FMT from QTQSF-treated mice attenuated renal injury and apoptosis, and increased IPA levels in DKD mice. **(A)** H&E and PAS staining showing renal histopathology **(B)** Masson staining and **(C)** quantitative analysis of renal fibrosis. **(D–H)** Western blots and densitometry of fibrosis- and apoptosis-related proteins, including α-sma, Vimentin, C-Cas3/Cas3 and Bax/Bcl-2. **(I–K)** Western blots and densitometry of podocyte markers including Synaptopodin and Nephrin. **(L,M)** IPA levels in serum and renal tissue. Data are presented as mean ± SEM (n = 3-5 per group). *P < 0.05, **P < 0.01, ***P < 0.001, ****P < 0.0001. Abbreviations: FMT, fecal microbiota transplantation; QTQSF, Qitu Qushi Formula; DKD, diabetic kidney disease; IPA, indole-3-propionic acid; H&E, hematoxylin and eosin; PAS, periodic acid-Schiff; C-Cas3/Cas3, cleaved caspase-3/total caspase-3; SEM, standard error of the mean; ABX, antibiotics.

To verify the microbiota-mediated effects of QTQSF, FMT experiments were performed in PGF db/db mice. Recipient mice in the H-QTQSF + FMT and ABX-H-QTQSF + FMT groups received fecal suspensions from the H-QTQSF and ABX-H-QTQSF donor groups, respectively (Figure 5A). After 10 weeks of FMT, mice in the H-QTQSF + FMT group showed a significant reduction in UACR compared with the ABX and ABX-H-QTQSF + FMT groups (P = 0.04 and P = 0.006, respectively) (Figures 5B,C). No significant differences were observed in SCr, body weight, and FBG (Figures 5D–F). Histological analyses (H&E, PAS) revealed improved glomerular architecture, reduced basement membrane thickening, and alleviated mesangial expansion in the H-QTQSF + FMT group (Figure 6A). Masson’s staining confirmed the lowest fibrosis scores in the H-QTQSF + FMT group (P < 0.005) (Figures 6B,C). Western blotting showed decreased α-sma and Vimentin expression and lower C-Cas3/Cas3 and Bax/Bcl-2 ratios (P < 0.05) (Figures 6D–H), while Synaptopodin and Nephrin expression levels increased (Figures 6I–K) in the H-QTQSF + FMT group. Notably, serum and renal IPA levels were markedly higher in the H-QTQSF + FMT group than in the other groups (serum P = 0.02; kidney P < 0.001) (Figures 6L,M). Collectively, these findings indicated that the intestinal flora of the H-QTQSF group had a therapeutic effect on PGF DKD mice, indicating that QTQSF exerted renoprotective effects partially by modulating gut microbiota and elevating gut-derived IPA levels.

### QTQSF-induced gut microbiota remodeling may contribute to its renoprotective effects in DKD mice

**FIGURE 7:**
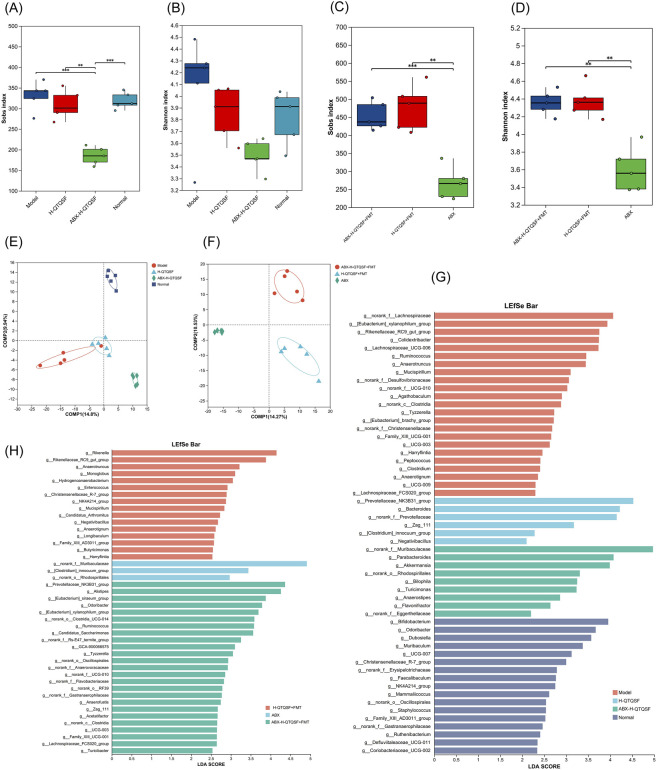
QTQSF intervention and FMT altered gut microbiota diversity and composition in DKD mice. **(A,B)** Alpha-diversity analysis of gut microbiota in the QTQSF intervention study, assessed by Sobs and Shannon indices. **(C,D)** Alpha-diversity analysis in the FMT study. **(E)** PLS-DA of gut microbiota following QTQSF intervention (LDA >2, P < 0.05). **(F)** PLS-DA of gut microbiota following FMT (LDA >2, P < 0.05). **(G,H)** LEfSe-derived LDA score histograms showing differential genera in the QTQSF intervention study and the FMT study, with significance thresholds of LDA >2 and P < 0.05; taxa containing “Unclassified” in their names were not displayed. Abbreviations: QTQSF, Qitu Qushi Formula; FMT, fecal microbiota transplantation; DKD, diabetic kidney disease; PLS-DA, partial least squares discriminant analysis; LDA, linear discriminant analysis; ABX, antibiotics.

16S rRNA sequencing was used to analyze gut microbiota following QTQSF intervention and FMT at the OUT level. In the QTQSF intervention study, the ABX-H-QTQSF group exhibited a significantly lower Sobs index compared to the other groups (Figure 7A). Although no statistical differences in Shannon diversity were found, the Model group had a higher Shannon index than the Normal group, and the H-QTQSF group’s index approached that of the Normal group, suggesting QTQSF may partially restore gut microbiota structure in DKD mice (Figure 7B). In the FMT study, both the Sobs and Shannon indices were significantly lower in the ABX group than in the other two groups, while no significant differences were observed between the ABX-H-QTQSF + FMT and H-QTQSF + FMT groups (Figures 7C,D). These results indicated that antibiotics intervention reduced gut microbiota richness and diversity, whereas FMT restored both. PLS-DA analysis confirmed distinct clustering of gut microbiota in both the QTQSF intervention (Figure 7E) and FMT studies (Figure 7F), with significant inter-group separation observed following 12 weeks of QTQSF intervention and 10 weeks of FMT. These findings suggest that both QTQSF intervention and FMT induced significant changes in gut microbiota composition in DKD mice.

**FIGURE 8:**
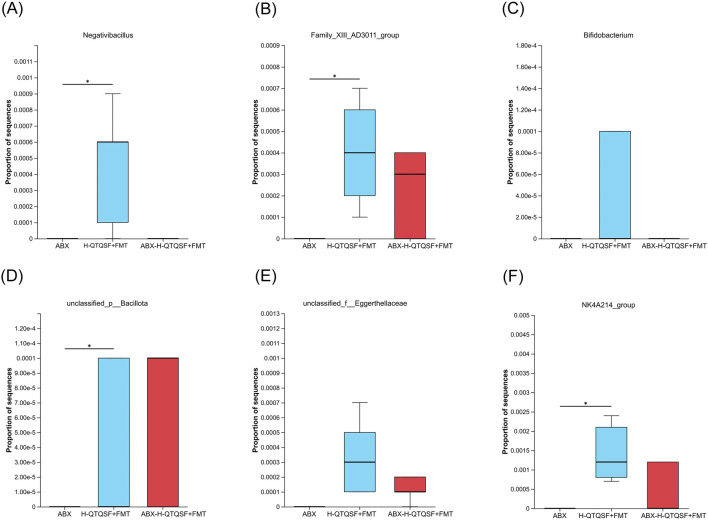
Differential gut microbial genera associated with FMT in DKD mice. **(A–F)** Relative abundances of shared dominant genera among ABX, H-QTQSF + FMT and ABX-H-QTQSF + FMT groups. Statistical analysis was performed using the Kruskal–Wallis H test on the genus level. Data are presented as mean ± SD. *P < 0.05, **P < 0.01, ***P < 0.001, ****P < 0.0001. Abbreviations: QTQSF, Qitu Qushi Formula; DKD, diabetic kidney disease; FMT, fecal microbiota transplantation; ABX, antibiotics.

LEfSe analysis identified 64 differential taxa in the QTQSF intervention study and 54 in the FMT experiment (LDA>2, P < 0.05) (Supplementary Table S5, S6; Figures 7G,H). Based on these results, *Negativibacillus, Family XIII AD3011 group, Bifidobacterium, unclassified_p__Bacillota, unclassified_f__Eggerthellaceae, and NK4A214 group* were found to be consistently enriched in the H-QTQSF + FMT group as well as in the H-QTQSF or Normal groups (Supplementary Table S7). Comparison of these genera across the ABX, H-QTQSF + FMT, and ABX-H-QTQSF + FMT groups revealed higher abundances of *Negativibacillus, Family XIII AD3011 group*, *unclassified_f__Eggerthellaceae*, and *NK4A214 group* in the H-QTQSF + FMT group, although not all differences reached statistical significance (Figures 8A–F). Collectively, these results indicated that QTQSF-induced modulation of gut microbial composition is likely associated with its renoprotective effects in DKD, supporting a microbiota-mediated mechanism of action.

### QTQSF-mediated renoprotection in DKD mice involved the IPA-Sirt1/FoxO1 signaling pathway

**FIGURE 9:**
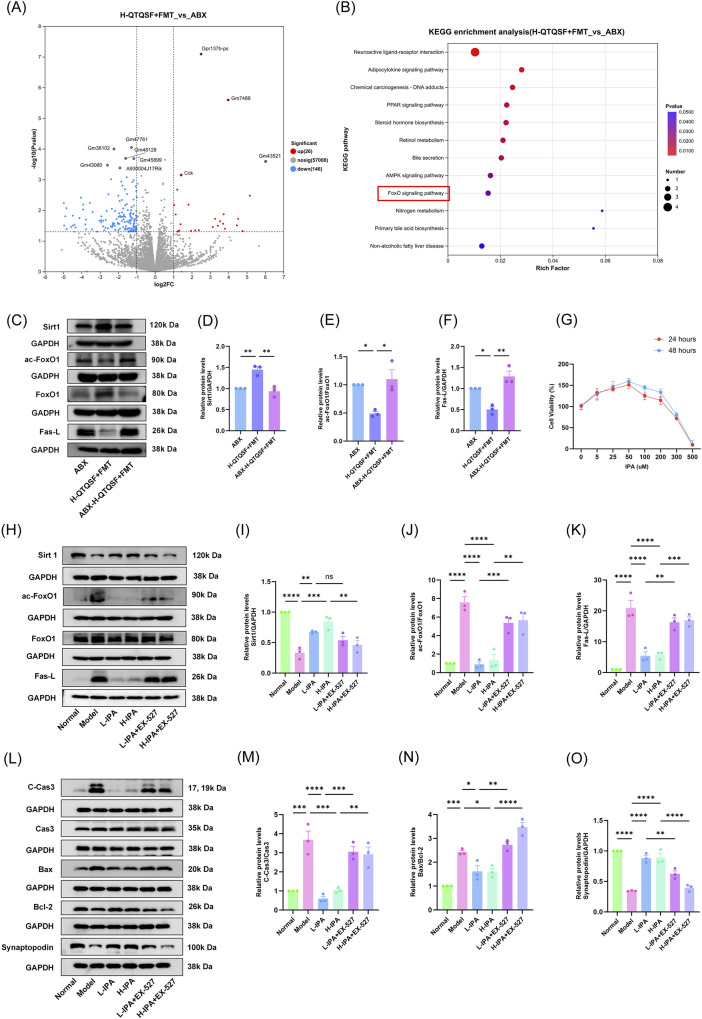
QTQSF-mediated renoprotection in DKD mice involved the IPA-Sirt1/FoxO1 signaling pathway, and IPA attenuated apoptosis *via* the Sirt1/FoxO1 pathway in MPC-5 cells. **(A)** Volcano plot showing differentially expressed genes in renal tissues between the H-QTQSF + FMT and ABX groups. **(B)** KEGG pathway enrichment analysis of differentially expressed genes. **(C–F)** Western blots and densitometry of Sirt1 expression, ac-FoxO1/FoxO1 ratio, and Fas-L expression in renal tissues (n = 3 per group). **(G)** Cell viability of MPC-5 cells treated with IPA for 24 h and 48 h (n = 6 per group). **(H–O)** Western blots and densitometry of Sirt1, ac-FoxO1/FoxO1 ratio, Fas-L, C-Cas-3/Cas-3, Bax/Bcl-2 ratio, and Synaptopodin in MPC-5 cells treated with IPA in the presence or absence of the Sirt1 inhibitor EX-527 (n = 3 per group). Data are presented as mean ± SEM. *P < 0.05, **P < 0.01, ***P < 0.001, ****P < 0.0001. Abbreviations: QTQSF, Qitu Qushi Formula; DKD, diabetic kidney disease; FMT, fecal microbiota transplantation; IPA, indole-3-propionic acid; KEGG, Kyoto Encyclopedia of Genes and Genomes; SEM, standard error of the mean; ABX, antibiotics, ac-FoxO1, acetyl-FoxO1, C-Cas-3/Cas-3, cleaved caspase-3/total caspase-3.

RNA-seq analysis was performed on kidney tissues from the H-QTQSF + FMT and ABX groups. This revealed 172 differentially expressed genes, including 26 upregulated and 146 downregulated genes (Figure 9A). KEGG pathway enrichment analysis revealed significant enrichment of the FoxO signaling pathway (Figure 9B). Our study further showed that this pathway was associated with altered expression of apoptosis-related genes, characterized by upregulation of Irs3 and downregulation of Fas-L (Supplementary Table S5). Given that IPA stabilizes Sirt1 by preventing its ubiquitin-proteasome degradation, and Sirt1 deacetylates FoxO1, we hypothesized that IPA may reduce renal apoptosis *via* the Sirt1-FoxO1-Fas-L axis. Western blot analysis confirmed this mechanism: Sirt1 expression was higher in the H-QTQSF + FMT group compared to the ABX and ABX-H-QTQSF + FMT groups (P = 0.005, P = 0.003), along with a reduced ac-FoxO1/FoxO1 ratio (P = 0.03, 0.01) and lower Fas-L expression (P = 0.007, <0.001) (Figures 9C–F). In conclusion, IPA derived from the gut microbiota after QTQSF treatment may exert nephroprotective effects by activating the Sirt1/FoxO1 pathway, thereby suppressing apoptosis.

### IPA attenuated apoptosis in MPC-5 cells *via* the Sirt1/FoxO1 pathway

Given the *in vivo* evidence that QTQSF modulated podocyte-associated proteins, we further investigated the underlying mechanisms in cultured podocytes. Cytotoxicity assays showed that MPC-5 cells did not exhibit cytotoxicity across IPA concentrations up to 200 μM at both 24 and 48 h. Notably, 25 μM and 50 μM achieved maximal viability and modestly promoted cell growth, and were thus selected for subsequent mechanistic studies (Figure 9G). Compared with the Model group, IPA treatment (L-IPA and H-IPA) upregulated Sirt1 expression, decreased the ac-FoxO1/FoxO1 ratio, and reduced Fas-L, C-Cas-3/Cas-3 and Bax/Bcl-2 ratios, indicating inhibition of apoptosis. Notably, IPA also increased Synaptopodin expression, suggesting a protective effect on podocyte integrity. However, the Sirt1 inhibitor EX-527 abolished these protective effects. In the L-IPA + EX-527 and H-IPA + EX-527 groups, Sirt1 and Synaptopodin expression declined, the ac-FoxO1/FoxO1 ratio increased, and apoptotic markers Fas-L, C-Cas-3/Cas-3 and Bax/Bcl-2 ratios were significantly upregulated (Figures 9H–O). Overall, these results demonstrated that IPA may protect MPC-5 cells from high-glucose-induced apoptosis through activation of the Sirt1/FoxO1 signaling pathway.

## Discussion

In this study, our findings suggested that QTQSF may alleviate DKD *via* a gut microbiota-derived IPA-Sirt1/FoxO1 regulatory axis. QTQSF reshapes the gut microbiota and elevates the tryptophan-derived metabolite IPA levels, which subsequently regulates renal Sirt1/FoxO1 signaling to suppress podocyte apoptosis to confer renal protection. These findings outline a microbiota-metabolite-signaling cascade that mechanistically links the clinical efficacy of QTQSF to its renal protective effects in DKD.

Previous research has identified alterations in amino acid metabolism as critical metabolic characteristics associated with the onset and progression of diabetes and DKD (Zhu et al., 2022). Among these alterations, tryptophan metabolism exerts bidirectional effects on DKD pathogenesis: activation of the kynurenine pathway exacerbates renal injury through oxidative stress, inflammation, and endothelial dysfunction, whereas gut microbiota-derived indole metabolites, such as IPA, confer significant renoprotective effects (Agus et al., 2018; Roager and Licht, 2018).

In the present study, we observed an increase in IPA levels following QTQSF treatment. As IPA is exclusively produced by gut microbiota *via* the tryptophan-indole pathway, this finding directly supports a close association between QTQSF intervention and gut microbial remodeling (Roager and Licht, 2018). Notably, *Cuscuta chinensis*, a key botanical drug of QTQSF, has been shown to alter gut microbial composition and relative abundance, while its major bioactive metabolite, *hyperoside*, may increase intestinal IPA levels through microbiota modulation (Hou et al., 2023; Zhao et al., 2023). Moreover, accumulating evidence indicated that other metabolites derived from the botanical drugs in QTQSF, including *polysaccharides* (e.g., *Astragalus, Atractylodes lancea*, and *Cordyceps cicadae*) and *triterpenoids (asiaticoside* and *asiatic acid* from *Centella asiatica)*, as well as representative *flavonoids* such as *nobiletin and hesperidin* and *phenolic acids* including *chlorogenic acid derivatives*, are capable of modulating gut microbial composition, intestinal barrier function, and host metabolic homeostasis (EEstruel-Amades et al., 2019; Wang et al., 2023; Kong et al., 2025; Ni et al., 2025; Wang et al., 2025; Zhao et al., 2026). Collectively, such microbiota remodeling may influence tryptophan metabolism and enhance the production of indole-derived metabolites, providing a plausible mechanistic explanation for the observed elevation of IPA following QTQSF treatment.

Notably, our identification of *Negativibacillus, Family XIII AD3011 group, Bifidobacterium, unclassified_p__Bacillota, unclassified_f__Eggerthellaceae, and NK4A214 group* as potential mediators of QTQSF’s renoprotective effects further supports this mechanistic framework. Some of these taxa have been shown to participate in tryptophan metabolism or to support the production of indole-related metabolites. For example, *Bifidobacterium* has been reported to generate indolelactic acid, a precursor of IPA, and may contribute to a metabolic environment that supports IPA production by other taxa such as *Clostridium and Peptostreptococcus* (Sinha et al., 2024). Evidence indicated that high-level IPA production is largely confined to a small group of obligate anaerobic clostridia, including *Clostridium sporogenes* and *Peptostreptococcus anaerobius*, within the phylum *Bacillota* (Schütz et al., 2025). Moreover, *Negativibacillus* (Dai et al., 2024), *Family XIII AD3011* group (Liu P. et al., 2024), *Bacillota* (Dong et al., 2025), and *NK4A214 group* (Liu et al., 2025) have been identified as critical regulators of microbial tryptophan metabolism. Together, these observations suggest that modulation of the gut microbiota–tryptophan–indole axis may represent a potential mechanism underlying the therapeutic effects of QTQSF.

The renoprotective effects of elevated IPA are well documented. Previous studies reported that higher serum IPA concentrations have been associated with a reduced risk of type 2 diabetes (T2D) (Morze et al., 2022; Qi et al., 2022), and improved renal outcomes in DKD, including negative correlations with FBG, HbA1c, and UACR, and positive correlations with eGFR. Moreover, IPA supplementation has been shown to attenuate albuminuria, enhance glomerular filtration, and ameliorate renal fibrosis, tubular edema, inflammation, and glomerular damage (Zeng et al., 2025). These findings align with IPA’s roles in kidney disease, such as reducing oxidative stress, alleviating inflammation, improving glucose metabolism, and stabilizing Sirt1 protein to promote mitochondrial biogenesis (Agus et al., 2018; Roager and Licht, 2018; Zeng et al., 2025).

In addition to IPA, several intermediate metabolites in the indole biosynthesis pathway, including indole-3-acetaldehyde, indoleacetic acid, and L-formylkynurenine, were identified in our untargeted metabolomic analysis. These metabolites may serve as markers of shifts in microbial tryptophan metabolism. Indole-3-acetaldehyde is a key intermediate in tryptophan conversion to indole derivatives, while indoleacetic acid represents an alternative metabolic branch (Roager and Licht, 2018). The observed increase in indole-3-acetaldehyde and decrease in indoleacetic acid suggest a metabolic shift towards IPA production. However, specific microbial genes or enzymes involved in this pathway were not assessed, highlighting the need for further investigation through metagenomic or metatranscriptomic approaches.

In this study, QTQSF treatment markedly reduced renal apoptosis, which may be mediated by the IPA–Sirt1/FoxO1 signaling pathway. IPA has been shown to stabilize Sirt1 by inhibiting its ubiquitin-proteasome degradation, thereby enhancing cellular resistance to apoptosis (Zeng et al., 2025). FoxO1, a key substrate of Sirt1, regulates apoptotic sensitivity in an acetylation-dependent manner. Ac-FoxO1 promotes pro-apoptotic gene expression (Caulfield and Lathem, 2014; Zhang et al., 2019), whereas Sirt1-mediated deacetylation protects against high glucose-induced podocyte apoptosis and enhances cell survival (Zhou et al., 2019).

The Sirt1/FoxO axis is increasingly recognized as a key renoprotective pathway modulated by TCM interventions. Previous studies have shown that metabolites derived from the botanical drugs in QTQSF can regulate Sirt1/FoxO-related signaling and confer renal benefits in DKD or related nephropathies. For example, *Astragalus polysaccharide* attenuates DKD through Sirt1/FoxO1-mediated suppression of apoptosis and enhancement of autophagy (Kempler and Körei, 2024), while *Atractylodes lancea* preserves podocyte function under metabolic stress *via* Sirt1 activation (Yang et al., 2023). In addition, representative metabolites identified in QTQSF, such as *formononetin and quercetin*, have been reported to modulate Sirt1-dependent signaling and FoxO1-mediated autophagic and mitochondrial pathways, whereas *asiaticoside* and its *aglycone asiatic acid* may further contribute to renoprotection by restoring podocyte autophagy and improving gut microbiota dysbiosis (Zhuang et al., 2020; Huang et al., 2022; Hou et al., 2025; Ni et al., 2025). These observations suggest that modulation of the Sirt1/FoxO pathway may contribute to the therapeutic effects of QTQSF; however, the potential involvement of gut microbiota-derived metabolites in this regulatory process remains insufficiently explored.

Our findings suggested that QTQSF is associated with gut microbiota remodeling and increased production of the tryptophan-derived metabolite IPA, which may potentially contribute to the regulation of the Sirt1/FoxO1 pathway. Depletion of gut microbiota markedly attenuated the renoprotective effects of QTQSF, whereas FMT partially restored therapeutic efficacy, supporting a central role for the gut microbiota in mediating QTQSF action. Mechanistically, elevated IPA levels were associated with increased renal Sirt1 expression, reduced FoxO1 acetylation, and attenuation of downstream pro-apoptotic signaling. While our findings support a role of the IPA–Sirt1/FoxO1 axis, additional pathways related to oxidative stress, inflammation, and mitochondrial function may also contribute. Collectively, these findings suggested that a gut microbiota–IPA–Sirt1/FoxO1 regulatory axis may represent a mechanism underlying QTQSF-mediated renoprotection and underscored the involvement of gut microbiota-derived metabolites in the therapeutic effects of TCM formulations in DKD.

Several limitations should be acknowledged. The lack of metagenomic sequencing limited the identification of microbial genes or enzymes involved in the conversion of tryptophan to IPA, thereby restricting a more detailed functional characterization of relevant microbial pathways. In addition, although comprehensive chemical profiling of QTQSF was conducted and the major metabolites were identified, the specific metabolites, or combinations of metabolites, responsible for modulating gut microbiota composition, IPA production, and coordinated regulation of the Sirt1/FoxO1 pathway remain to be elucidated. These issues represent important directions for future investigation and are currently being addressed in ongoing studies.

In conclusion, our findings suggested that QTQSF may alleviate DKD through a mechanism associated with gut microbiota modulation and increased production of the tryptophan-derived metabolite IPA, potentially involving regulation of the Sirt1/FoxO1 signaling pathway and attenuation of podocyte apoptosis. These results provide insight into a possible microbiota–metabolite–signaling axis underlying the therapeutic effects of QTQSF and highlight the potential role of gut microbiota-derived metabolites in DKD management.

## Data Availability

The sequencing data generated in this study have been deposited in the NCBI Sequence Read Archive (SRA). Human and mouse gut microbiota 16S rRNA sequencing data are available under BioProject accession PRJNA1468911, and mouse kidney transcriptome sequencing data are available under BioProject accession PRJNA1469266. Other data supporting the findings of this study are available from the corresponding author upon reasonable request.

## Glossary

DKD: diabetic kidney disease
ESRD: end-stage renal disease
QTQSF: Qitu Qushi Formula
TCM: traditional Chinese medicine
DAPA: dapagliflozin
FMT: fecal microbiota transplantation
IPA: indole-3-propionic acid
Sirt1: sirtuin 1
eGFR: estimated glomerular filtration rate
SGLT2: sodium-glucose cotransporter 2
GLP-1: glucagon-like peptide-1
UHPLC-MS/MS: ultra-high-performance liquid chromatography coupled with tandem mass spectrometry
SPF: specific pathogen-free
ABX: antibiotics
FBG: fasting blood glucose
UACR: urinary albumin-to-creatinine ratio
SCr: serum creatinine
HbA1c: glycated hemoglobin A1c
H&E: hematoxylin-eosin
PAS: periodic acid–Schiff
Cas-3: caspase-3
C-Cas-3: cleaved caspase-3
ac-FoxO1: acetylated FoxO1
CCK-8: Cell Counting Kit-8
OTUs: Operational taxonomic units
PLS-DA: partial least squares discriminant analysis
LEfSe: linear discriminant analysis effect size
LDA: linear discriminant analysis
QC: quality control
OPLS-DA: orthogonal partial least squares discriminant analysis
KEGG: Kyoto Encyclopedia of Genes and Genomes
MRM: multiple reaction monitoring
PCA: Principal Component Analysis
VIP: variable importance in projection
IQR: interquartile range
