# Supplementary figures and images for: Qitu qushi formula ameliorates diabetic kidney disease potentially through gut microbiota-derived indole-3-propionic Acid–Mediated regulation of the Sirt1/FoxO1 pathway
Source: Front Pharmacol. 2026 Jun 2;17:1802567. doi: 10.3389/fphar.2026.1802567 (PMC13269076; doi:10.3389/fphar.2026.1802567)

(A)

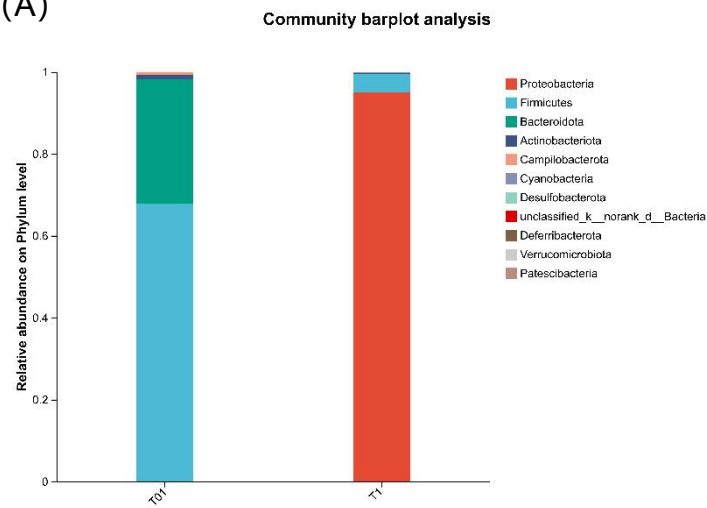

(B)

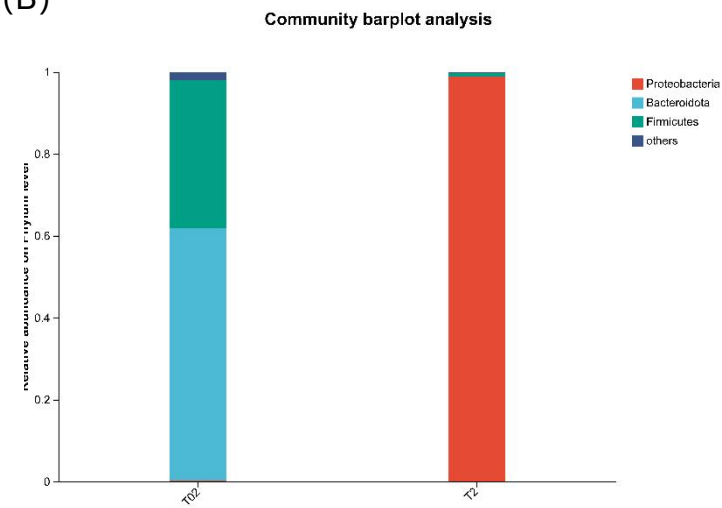

Supplement: Supplementary file 1 [file DataSheet2.pdf]

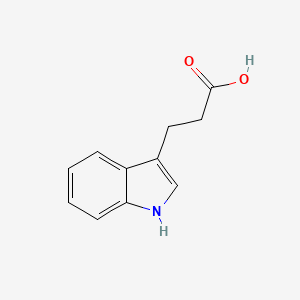

Supplement: Supplementary file 2 [file Image1.tiff]

(A)

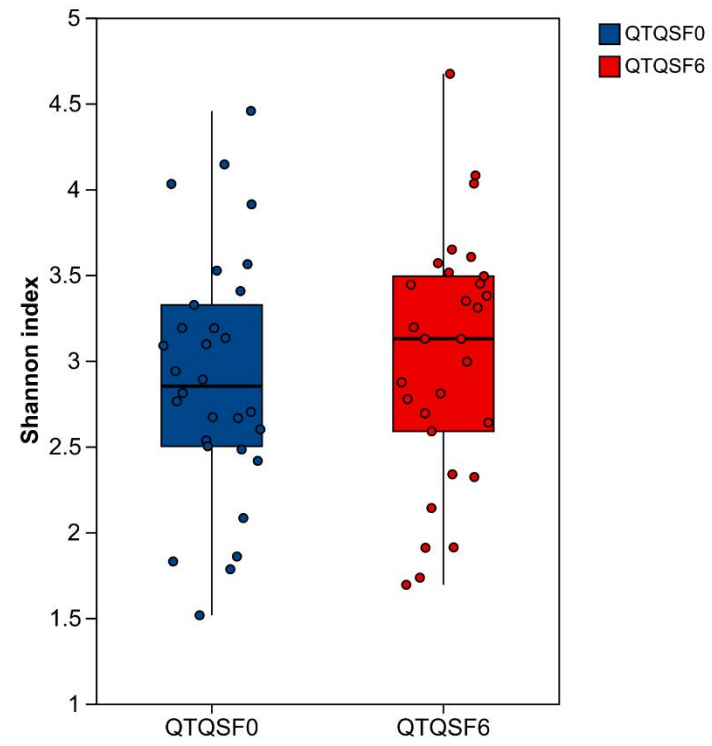

(B)

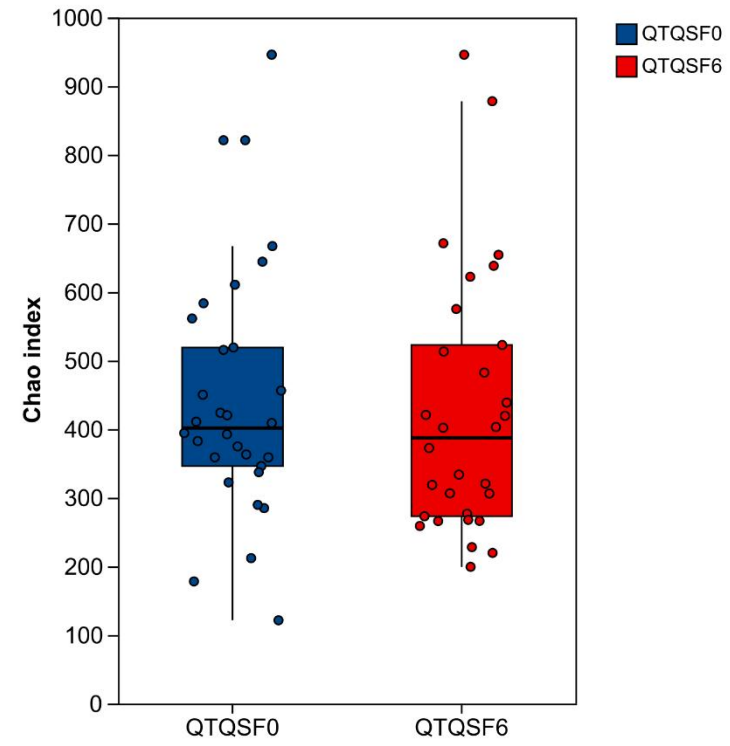

Supplement: Supplementary file 5 [file DataSheet3.pdf]

(A)

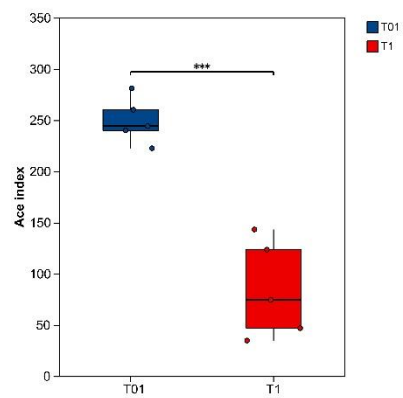

(B)

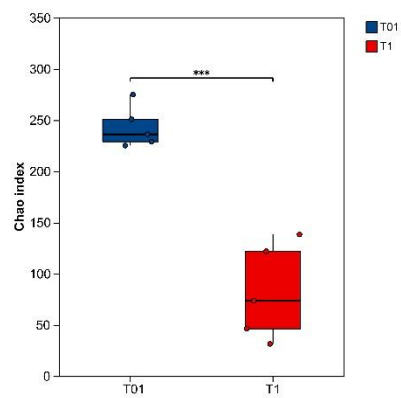

(C)

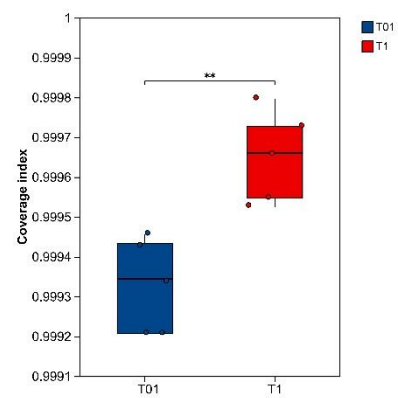

(D)

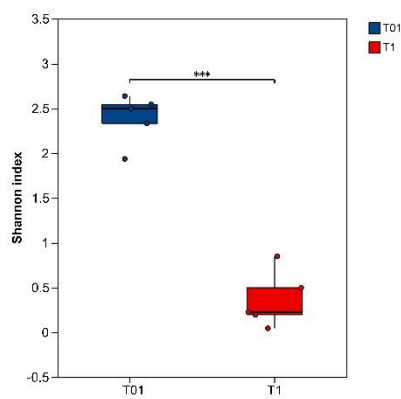

(E)

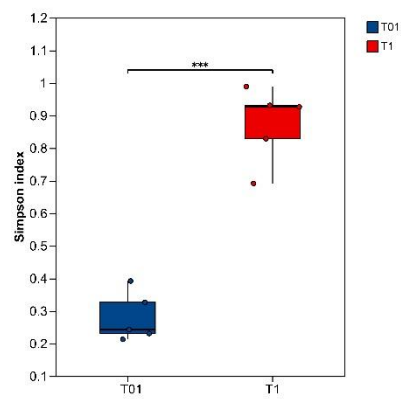

(F)

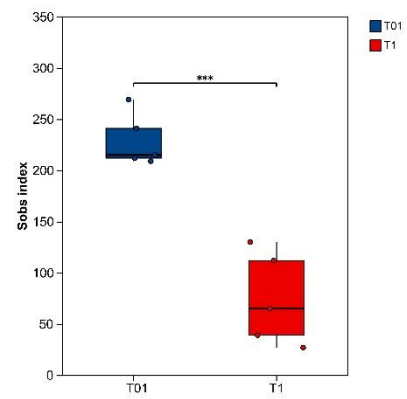

(G)

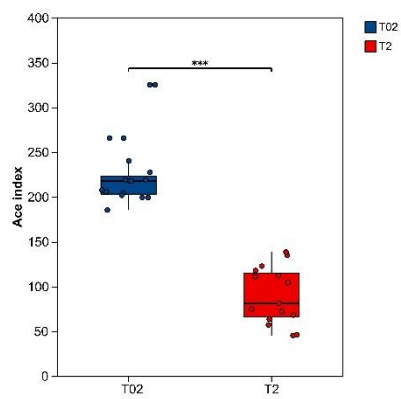

(H)

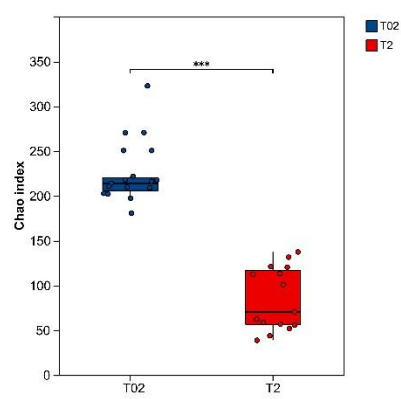

(I)

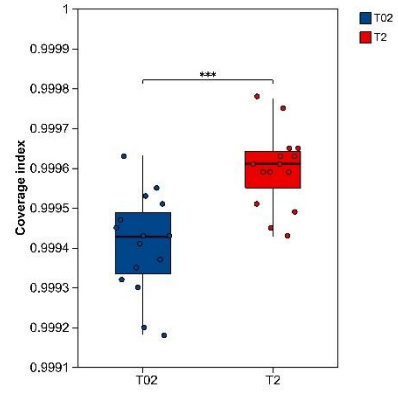

(J)

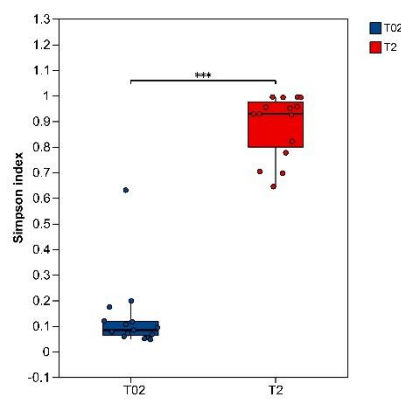

(K)

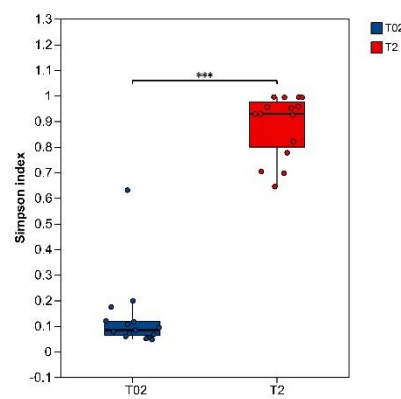

(L)

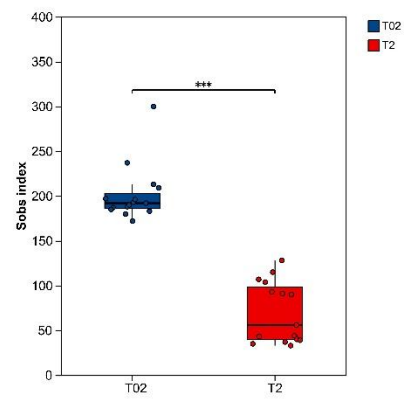

Supplement: Supplementary file 7 [file DataSheet1.pdf]

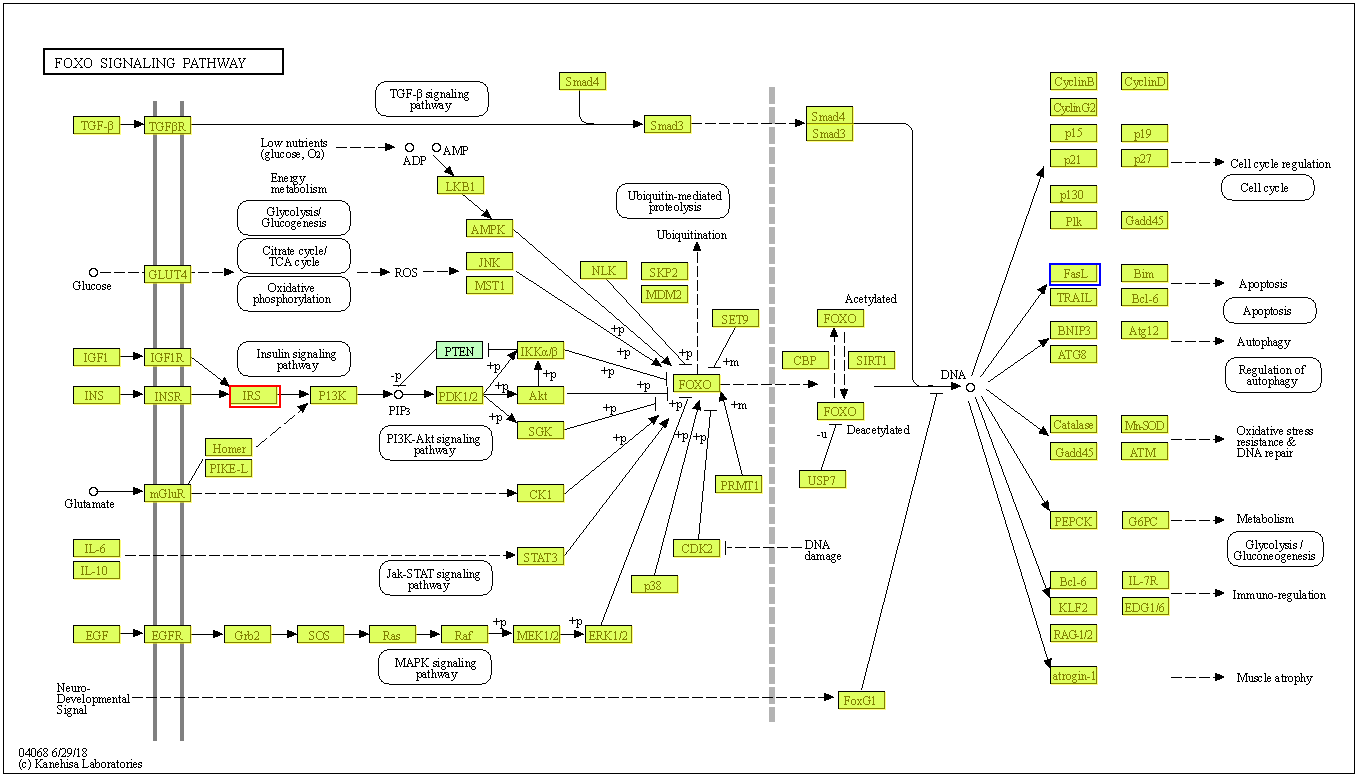

Supplement: Supplementary file 12 [file Image2.tiff]
