# Supplementary material for: Qitu qushi formula ameliorates diabetic kidney disease potentially through gut microbiota-derived indole-3-propionic Acid–Mediated regulation of the Sirt1/FoxO1 pathway
Source: Front Pharmacol. 2026 Jun 2;17:1802567. doi: 10.3389/fphar.2026.1802567 (PMC13269076; doi:10.3389/fphar.2026.1802567)
Supplement: Supplementary file 3 [file Table1.docx]

Table S1 Identification of compounds in QTQSF granules by UHPLC–HRMS analysis.

| Compound Name | Molecular Formula | Molecular  Weight (g·mol⁻¹) | Retention  Time(min) | Match Score | Peak Area | Relative  Content (%) |
| --- | --- | --- | --- | --- | --- | --- |
| Amygdalin | C20 H27 N O11 | 457.15825 | 20.348 | 92.3 | 8783621955 | 9.034 |
| Hesperidin | C28 H34 O15 | 610.18939 | 23.688 | 90.2 | 8513730987 | 8.756 |
| Citric acid | C6 H8 O7 | 192.02696 | 1.845 | 89.8 | 7884089131 | 8.109 |
| Stachydrine | C7 H13 N O2 | 143.09473 | 1.7 | 75 | 6456825650 | 6.641 |
| L-Proline | C5 H9 N O2 | 115.06341 | 1.71 | 83.1 | 5494744834 | 5.651 |
| Nobiletin | C21 H22 O8 | 402.13141 | 33.788 | 87.5 | 4370053424 | 4.495 |
| Sucrose | C12 H22 O11 | 342.11619 | 1.718 | 93.8 | 4306980395 | 4.43 |
| Narirutin | C27 H32 O14 | 580.17896 | 23.123 | 88.1 | 3732195661 | 3.839 |
| Hesperetin | C16 H14 O6 | 302.07862 | 23.686 | 89.3 | 2984837673 | 3.07 |
| Calycosin | C16 H12 O5 | 284.06821 | 26.827 | 89.8 | 2881561840 | 2.964 |
| Formononetin | C16 H12 O4 | 268.07341 | 31.104 | 86.5 | 2493016213 | 2.564 |
| Asiaticoside | C48 H78 O19 | 958.51302 | 25.992 | 81.8 | 2383555286 | 2.451 |
| Tangeretin | C20 H20 O7 | 372.12095 | 35.734 | 86 | 2260338874 | 2.325 |
| Cryptochlorogenic acid | C16 H18 O9 | 354.09501 | 20.159 | 92.3 | 2009372690 | 2.067 |
| Isochlorogenic acid C | C25 H24 O12 | 516.12637 | 23.944 | 92.9 | 1661481286 | 1.709 |
| Isochlorogenic acid B | C25 H24 O12 | 516.12633 | 23.14 | 90.1 | 1414706015 | 1.455 |
| 1,3-Dicaffeoylquinic acid | C25 H24 O12 | 516.12637 | 21.18 | 92.8 | 1389145053 | 1.429 |
| Calycosin-7-O-β-D-glucoside | C22 H22 O10 | 446.12124 | 22.372 | 87.3 | 1380323474 | 1.42 |
| Adenosine | C10 H13 N5 O4 | 267.09684 | 11.795 | 90.6 | 1336995553 | 1.375 |
| Betaine | C5 H11 N O2 | 117.079 | 1.62 | 84.8 | 1186909231 | 1.221 |
| Hyperoside | C21 H20 O12 | 464.0956 | 22.643 | 92.5 | 1094592381 | 1.126 |
| 3,5-Dicaffeoylquinic acid | C25 H24 O12 | 516.12636 | 23.522 | 90.1 | 1009981999 | 1.039 |
| L-Leucine | C6 H13 N O2 | 131.09471 | 4.951 | 75.7 | 996122880.2 | 1.025 |
| L-Phenylalanine | C9 H11 N O2 | 165.07913 | 13.536 | 79.2 | 960813585.2 | 0.988 |
| Trigonelline | C7 H7 N O2 | 137.04771 | 1.695 | 88.6 | 884380589 | 0.91 |
| Ononin | C22 H22 O9 | 430.12627 | 25.043 | 87.2 | 796668863.7 | 0.819 |
| Isosinensetin | C20 H20 O7 | 372.1206 | 30.384 | 90.8 | 686015080.6 | 0.706 |
| Naringenin chalcone | C15 H12 O5 | 272.06815 | 23.123 | 84.5 | 682398422.3 | 0.702 |
| Sinensetin | C20 H20 O7 | 372.32059 | 32.044 | 87.4 | 650809804.6 | 0.669 |
| Quercetin 3-O-β-D-Glucuronide | C21 H18 O13 | 478.07466 | 22.714 | 89.8 | 637268636.1 | 0.655 |
| Protocatechualdehyde | C7 H6 O3 | 138.03155 | 19.559 | 83.8 | 634945095.6 | 0.653 |
| Quercetin | C15 H10 O7 | 302.04244 | 26.865 | 91.8 | 612279166.4 | 0.63 |
| 6-Demethoxytangeretin | C19 H18 O6 | 342.11021 | 32.125 | 89.5 | 600321169 | 0.617 |
| 5-Hydroxymethylfurfural | C6 H6 O3 | 126.03179 | 18.559 | 78.5 | 582726725.3 | 0.599 |
| Quinic acid | C7 H12 O6 | 192.06325 | 20.103 | 89 | 552769653.5 | 0.569 |
| Morin | C15 H10 O7 | 302.04248 | 22.652 | 89 | 551524614.1 | 0.567 |
| p-Coumaric acid | C9 H8 O3 | 164.04751 | 5.77 | 79.8 | 544821094.4 | 0.56 |
| Manninotriose | C18 H32 O16 | 504.16917 | 1.706 | 80.8 | 526111663.9 | 0.541 |
| Didymin | C28 H34 O14 | 594.19486 | 26.094 | 89.3 | 523558300.6 | 0.538 |
| Isoguanosine | C10 H13 N5 O5 | 283.09164 | 14.345 | 84.7 | 520490634.1 | 0.535 |
| Kaempferol | C15 H10 O6 | 286.04764 | 29.177 | 93.7 | 518115640.5 | 0.533 |
| Quillaic acid | C30 H46 O5 | 486.33397 | 24.854 | 71.4 | 509935069.5 | 0.524 |
| Nicotinic acid | C6 H5 N O2 | 123.03211 | 3.028 | 81 | 454103325.4 | 0.467 |
| Mannitol | C6 H14 O6 | 182.07886 | 1.564 | 87.1 | 432944122.4 | 0.445 |
| Azelaic acid | C9 H16 O4 | 188.10481 | 24.316 | 76.5 | 431654750.6 | 0.444 |
| Uridine | C9 H12 N2 O6 | 244.06945 | 6.013 | 92.7 | 406484329.3 | 0.418 |
| Glabrolide | C30 H44 O4 | 468.32363 | 24.854 | 72.6 | 405878653.3 | 0.417 |
| Methylnissolin-3-O-glucoside | C23 H26 O10 | 462.15237 | 25.88 | 87.8 | 364780224.8 | 0.375 |
| Caffeic acid | C9 H8 O4 | 180.03669 | 20.856 | 87.1 | 332458733.8 | 0.342 |
| Astragaloside IV | C41 H68 O14 | 784.96651 | 30.252 | 84.2 | 329471657.5 | 0.339 |
| L-Threonine | C4 H9 N O3 | 119.05826 | 1.54 | 82.5 | 311709702.1 | 0.321 |
| Isosakuranetin | C16 H14 O5 | 286.0838 | 26.091 | 88.7 | 288682816.1 | 0.297 |
| Astragalin | C21 H20 O11 | 448.10046 | 23.534 | 84 | 283322982.7 | 0.291 |
| 6-Hydroxyindole | C8 H7 N O | 133.05287 | 18.496 | 80.1 | 255221991.2 | 0.262 |
| Vicenin II | C27 H30 O15 | 594.15872 | 20.666 | 87.5 | 249532426.7 | 0.257 |
| Scutellarin | C21 H18 O12 | 462.33717 | 23.573 | 78.1 | 243608835.9 | 0.251 |
| 6''-O-Acetylglycitin | C24 H24 O11 | 488.1313 | 24.817 | 75.6 | 221843853.9 | 0.228 |
| 4-Hydroxybenzoic acid | C7 H6 O3 | 138.03154 | 24.953 | 85 | 194911086.4 | 0.2 |
| Isomucronulatol 7-O-glucoside | C23 H28 O10 | 464.16827 | 26.248 | 88.5 | 184271521.3 | 0.19 |
| Eriocitrin | C27 H32 O15 | 596.17444 | 22.155 | 75.4 | 174038358.8 | 0.179 |
| Astragaloside III | C41 H68 O14 | 784.461 | 30.54 | 85.2 | 171916671 | 0.177 |
| Rutin | C27 H30 O16 | 610.15365 | 22.29 | 91.3 | 164376268.8 | 0.169 |
| Stachyose | C24 H42 O21 | 666.22209 | 1.714 | 88.5 | 143068124.4 | 0.147 |
| Dehydrocostus lactone | C15 H18 O2 | 230.13055 | 35.282 | 84.4 | 142721790.2 | 0.147 |
| Diosmin | C28 H32 O15 | 608.17416 | 23.598 | 81.5 | 141404445.5 | 0.145 |
| Ferulic acid | C10 H10 O4 | 194.05801 | 20.071 | 79.7 | 138834835.2 | 0.143 |
| 5-O-Demethylnobiletin | C20 H20 O8 | 388.11571 | 37.479 | 90.1 | 132312641.4 | 0.136 |
| Protocatechuic acid | C7 H6 O4 | 154.02653 | 17.982 | 84.4 | 129316912 | 0.133 |
| Maltopentaose | C30 H52 O26 | 828.72696 | 1.723 | 89.8 | 119797664.5 | 0.123 |
| Abscisic acid | C15 H20 O4 | 264.13599 | 26.269 | 80.6 | 114657752.7 | 0.118 |
| Esculetin | C9 H6 O4 | 178.02654 | 20.798 | 73.6 | 110471645.3 | 0.114 |
| Isorhamnetin-3-O-nehesperidine | C28 H32 O16 | 624.16918 | 23.562 | 83.6 | 97831595.62 | 0.101 |
| Isorhamnetin | C16 H12 O7 | 316.05812 | 23.651 | 83.6 | 94121529.06 | 0.097 |
| Dehydrotrametenolic acid | C30 H46 O3 | 454.34447 | 30.255 | 77.4 | 93396977.98 | 0.096 |
| Asiatic acid | C30 H48 O5 | 488.34969 | 25.994 | 72.3 | 93113183.01 | 0.096 |
| Isosakuranin | C22 H24 O10 | 448.13664 | 26.091 | 86.1 | 81077941.11 | 0.083 |
| Isofraxidin | C11 H10 O5 | 222.05272 | 23.357 | 79.6 | 78570382.59 | 0.081 |
| Isoastragaloside I | C45 H72 O16 | 869.06299 | 36.211 | 85.3 | 75499819.23 | 0.078 |
| Curcumol | C15 H24 O2 | 236.17757 | 28.817 | 78.3 | 74656153.72 | 0.077 |
| Limonin | C26 H30 O8 | 470.48074 | 22.543 | 76.9 | 66147678.88 | 0.068 |
| Scoparone | C11 H10 O4 | 206.05799 | 25.468 | 77 | 61774088.87 | 0.064 |
| α-Linolenic acid | C18 H30 O2 | 278.2245 | 37.051 | 80.5 | 56706704.97 | 0.058 |
| o-Veratraldehyde | C9 H10 O3 | 166.06294 | 26.252 | 76.7 | 55953574.2 | 0.058 |
| Gallic acid | C7 H6 O5 | 170.02145 | 8.325 | 80.1 | 54642504.66 | 0.056 |
| Neohesperidin | C28 H34 O15 | 610.18939 | 24.671 | 89.3 | 53905414.06 | 0.055 |
| Diosmetin | C16 H12 O6 | 300.06314 | 29.653 | 74.3 | 53525233.14 | 0.055 |
| Adenine | C5 H5 N5 | 135.05441 | 11.815 | 72.4 | 52894205.25 | 0.054 |
| Atractylenolide II | C15 H20 O2 | 232.14646 | 19.178 | 79.3 | 49080686.31 | 0.05 |
| Vicenin III | C26 H28 O14 | 564.1481 | 21.335 | 80.5 | 47155843.23 | 0.048 |
| Kaempferol-3-O-rutinoside | C27 H30 O15 | 594.16537 | 23.077 | 84.7 | 44774744.89 | 0.046 |
| Isomeranzin | C15 H16 O4 | 278.1154 | 25.302 | 81.7 | 42812354.1 | 0.044 |
| Atractylenolide III | C15 H20 O3 | 248.1411 | 35.28 | 81.5 | 40455370.87 | 0.042 |
| Pinoresinol 4-O-glucoside | C26 H32 O11 | 520.19432 | 23.231 | 81.6 | 37602325.09 | 0.039 |
| Roburic acid | C30 H48 O2 | 408.33915 | 32.296 | 70.7 | 34358312.46 | 0.035 |
| Artemisinin | C15 H22 O5 | 282.14678 | 19.587 | 73.2 | 34115564.32 | 0.035 |
| Apigenin | C15 H10 O5 | 270.05267 | 28.792 | 82.4 | 34002794.36 | 0.035 |
| 2-Hydroxy-4-methoxybenzaldehyde | C8 H8 O3 | 152.04738 | 22.442 | 80 | 29904391.98 | 0.031 |
| Methyl hexadecanoate | C17 H34 O2 | 316.26124 | 39.55 | 81.8 | 29105088.95 | 0.03 |
| Artemetin | C20 H20 O8 | 388.11571 | 34.324 | 85.4 | 28837651.91 | 0.03 |
| Narcissoside | C28 H32 O16 | 624.16924 | 23.206 | 82.8 | 28786561.83 | 0.03 |
| Linarin | C28 H32 O14 | 592.17943 | 25.799 | 78.4 | 28013582.66 | 0.029 |
| Pedunculoside | C36 H58 O10 | 696.40846 | 28.675 | 71.3 | 27880498.91 | 0.029 |
| Baicalin | C21 H18 O11 | 446.08476 | 24.999 | 70.1 | 27524981.88 | 0.028 |
| Eriodictyol | C15 H12 O6 | 288.06322 | 22.158 | 79.2 | 27109130.92 | 0.028 |
| Sibiricose A5 | C22 H30 O14 | 518.16356 | 20.863 | 82.4 | 26160871.45 | 0.027 |
| Apigenin 7-O-(2G-rhamnosyl)gentiobioside | C33 H40 O19 | 740.21644 | 20.931 | 74.6 | 25711819.63 | 0.026 |
| Artemisinic acid | C15 H22 O2 | 252.17242 | 21.588 | 81.2 | 23148995.36 | 0.024 |
| Chrysosplenetin B | C19 H18 O8 | 374.10016 | 34.142 | 76.2 | 22342628.25 | 0.023 |
| Hydroxygenkwanin | C16 H12 O6 | 300.06314 | 29.227 | 82 | 22262200.62 | 0.023 |
| Liquiritigenin | C15 H12 O4 | 256.07341 | 26.413 | 81.6 | 21110825.82 | 0.022 |
| Gardenin B | C19 H18 O7 | 358.10506 | 29.475 | 77 | 20786982.1 | 0.021 |
| Isoalantolactone | C15 H20 O2 | 232.14642 | 23.279 | 77.1 | 20435231.44 | 0.021 |
| Parthenolide | C15 H20 O3 | 248.14113 | 33.126 | 82.6 | 20284284.22 | 0.021 |
| 2-Adamantanone | C10 H14 O | 150.10451 | 29.752 | 70.6 | 19816939.53 | 0.02 |
| Paeoniflorin | C23 H28 O11 | 480.48959 | 21.692 | 70.1 | 18810473.41 | 0.019 |
| Orientin | C21 H20 O11 | 448.10065 | 21.771 | 79.5 | 17232436.51 | 0.018 |
| Echinocystic acid | C30 H48 O4 | 472.35527 | 30.533 | 70 | 17178873.9 | 0.018 |
| α-Cyperone | C15 H22 O | 236.1776 | 30.721 | 79 | 17066620.46 | 0.018 |
| Vitexin | C21 H20 O10 | 432.10571 | 22.446 | 76.6 | 17059986.76 | 0.018 |
| Senkyunolide A | C12 H16 O2 | 192.11527 | 40.956 | 71.1 | 15383372.81 | 0.016 |
| Astragaloside II | C43 H70 O15 | 826.47118 | 33.816 | 83.8 | 13479419.93 | 0.014 |
| Pinocembrin | C15 H12 O4 | 256.07326 | 25.867 | 79.3 | 12563723.57 | 0.013 |
| Quercetin 7-rhamnoside | C21 H20 O11 | 448.10048 | 24.763 | 82 | 12522779.3 | 0.013 |
| Biochanin A | C16 H12 O5 | 284.06823 | 34.699 | 81.8 | 11867829.88 | 0.012 |
| Arglabin | C15 H18 O3 | 246.12543 | 30.838 | 73.8 | 9038320.698 | 0.009 |
| Wogonoside | C22 H20 O11 | 460.10052 | 27.062 | 70.1 | 8904009.136 | 0.009 |
| Curdione | C15 H24 O2 | 236.17752 | 28.17 | 83.4 | 8745661.916 | 0.009 |
| Rosmarinic acid | C18 H16 O8 | 360.0847 | 24.25 | 70.7 | 7387357.61 | 0.008 |
| Wogonin | C16 H12 O5 | 284.06828 | 33.502 | 75.7 | 7054795.292 | 0.007 |
| 3,5-Dimethoxy-4-hydroxybenzaldehyde | C9 H10 O4 | 182.05823 | 27.25 | 74.4 | 3439063.173 | 0.004 |
