# Supplementary material for: Qitu qushi formula ameliorates diabetic kidney disease potentially through gut microbiota-derived indole-3-propionic Acid–Mediated regulation of the Sirt1/FoxO1 pathway
Source: Front Pharmacol. 2026 Jun 2;17:1802567. doi: 10.3389/fphar.2026.1802567 (PMC13269076; doi:10.3389/fphar.2026.1802567)
Supplement: Supplementary file 4 [file Table7.docx]

Table S7 Shared dominant gut microbiota between the H-QTQSF+FMT group as well as in the H-QTQSF or Normal groups of the QTQSF intervention study (LDA>2, P<0.05).

| Differential Taxa | Shared group | LDA Score | P value |
| --- | --- | --- | --- |
| g__Negativibacillus | H-QTQSF | 2.26 | 0.02 |
| g__Family_XIII_AD3011_group | Normal | 2.5 | 0 |
| g__Bifidobacterium | Normal | 3.94 | 0 |
| g__unclassified_p__Bacillota | H-QTQSF | 2.56 | 0.04 |
| g__unclassified_f__Eggerthellaceae | H-QTQSF | 2.23 | 0.03 |
| g__NK4A214_group | Normal | 2.75 | 0 |

Abbreviations: QTQSF, Qitu Qushi Formula; FMT, fecal microbiota transplantation, LDA, linear discriminant analysis; .
