# Supplementary material for: Qitu qushi formula ameliorates diabetic kidney disease potentially through gut microbiota-derived indole-3-propionic Acid–Mediated regulation of the Sirt1/FoxO1 pathway
Source: Front Pharmacol. 2026 Jun 2;17:1802567. doi: 10.3389/fphar.2026.1802567 (PMC13269076; doi:10.3389/fphar.2026.1802567)
Supplement: Supplementary file 6 [file Table2.docx]

Table S2 Changes in fecal DNA concentration during the establishment of the PGF mouse model.

| Experiments | Before modeling,  median (IQR) | After modeling, median (IQR) | P value |
| --- | --- | --- | --- |
| QTQSF intervention | 8.300 (8.150-10.250) | 1.600 (1.450-2.150) | 0.043 |
| FMT | 14.840 (11.690-32.030) | 2.360 (2.110-2.620) | 0.001 |

Abbreviations: PGF, pseudo-germ-free; IQR, interquartile range; QTQSF, Qitu Qushi Formula; FMT, fecal microbiota transplantation.
