# Supplementary material for: Qitu qushi formula ameliorates diabetic kidney disease potentially through gut microbiota-derived indole-3-propionic Acid–Mediated regulation of the Sirt1/FoxO1 pathway
Source: Front Pharmacol. 2026 Jun 2;17:1802567. doi: 10.3389/fphar.2026.1802567 (PMC13269076; doi:10.3389/fphar.2026.1802567)
Supplement: Supplementary file 8 [file Table5.docx]

Table S5 Differentially abundant gut microbial taxa in mice following Qitu Qushi Formula intervention (LDA>2, P<0.05).

| Differential Taxa | Predominant group | LDA Score | P value |
| --- | --- | --- | --- |
| g__norank_o__Oscillospirales | Normal | 2.59 | 0.01 |
| g__unclassified_f__Anaerovoracaceae | Normal | 3.58 | 0 |
| g__norank_f__Gastranaerophilaceae | Normal | 2.43 | 0 |
| g__Family_XIII_AD3011_group | Normal | 2.5 | 0 |
| g__Staphylococcus | Normal | 2.32 | 0.02 |
| g__UCG-007 | Normal | 2.93 | 0.02 |
| g__Bifidobacterium | Normal | 3.94 | 0 |
| g__Odoribacter | Normal | 3.72 | 0.01 |
| g__Christensenellaceae_R-7_group | Normal | 2.99 | 0.03 |
| g__Coriobacteriaceae_UCG-002 | Normal | 2.54 | 0 |
| g__Dubosiella | Normal | 3.62 | 0 |
| g__Faecalibaculum | Normal | 2.7 | 0 |
| g__norank_f__Erysipelotrichaceae | Normal | 2.79 | 0.01 |
| g__Mammaliicoccus | Normal | 2.72 | 0.02 |
| g__unclassified_f__Prevotellaceae | Normal | 2.72 | 0 |
| g__Defluviitaleaceae_UCG-011 | Normal | 2.22 | 0.03 |
| g__Muribaculum | Normal | 3.36 | 0.01 |
| g__NK4A214_group | Normal | 2.75 | 0 |
| g__Ruthenibacterium | Normal | 2.35 | 0.01 |
| g__norank_f__UCG-010 | Model | 3.05 | 0.01 |
| g__Clostridium | Model | 2.35 | 0.02 |
| g__[Eubacterium]_xylanophilum_group | Model | 3.91 | 0.02 |
| g__UCG-003 | Model | 2.67 | 0.04 |
| g__Agathobaculum | Model | 2.9 | 0.03 |
| g__Mucispirillum | Model | 3.13 | 0 |
| g__norank_f__Christensenellaceae | Model | 2.69 | 0 |
| g__Family_XIII_UCG-001 | Model | 2.68 | 0 |
| g__Lachnospiraceae_FCS020_group | Model | 2.34 | 0.02 |
| g__Peptococcus | Model | 2.4 | 0.03 |
| g__Rikenellaceae_RC9_gut_group | Model | 3.77 | 0 |
| g__Tyzzerella | Model | 2.67 | 0.03 |
| g__Harryflintia | Model | 2.44 | 0 |
| g__Ruminococcus | Model | 3.4 | 0.05 |
| g__norank_f__Desulfovibrionaceae | Model | 3.05 | 0.02 |
| g__Anaerotignum | Model | 2.36 | 0.01 |
| g__Lachnospiraceae_UCG-006 | Model | 3.85 | 0.03 |
| g__norank_c__Clostridia | Model | 2.91 | 0.01 |
| g__UCG-009 | Model | 2.22 | 0.02 |
| g__unclassified_f__Ruminococcaceae | Model | 3.08 | 0.04 |
| g__Anaerotruncus | Model | 3.57 | 0.04 |
| g__norank_f__Lachnospiraceae | Model | 4.08 | 0.03 |
| g__[Eubacterium]_brachy_group | Model | 2.73 | 0.01 |
| g__Colidextribacter | Model | 3.81 | 0.02 |
| g__Negativibacillus | H-QTQSF | 2.26 | 0.02 |
| g__[Clostridium]_innocuum_group | H-QTQSF | 2.58 | 0.03 |
| g__unclassified_p__Bacillota | H-QTQSF | 2.56 | 0.04 |
| g__Prevotellaceae_NK3B31_group | H-QTQSF | 4.51 | 0 |
| g__unclassified_f__Eggerthellaceae | H-QTQSF | 2.23 | 0.03 |
| g__unclassified_f__Sutterellaceae | H-QTQSF | 2.5 | 0.05 |
| g__Bacteroides | H-QTQSF | 4.28 | 0.01 |
| g__Intestinimonas | H-QTQSF | 2.23 | 0.05 |
| g__norank_f__Prevotellaceae | H-QTQSF | 4.21 | 0.01 |
| g__Zag_111 | H-QTQSF | 3.25 | 0 |
| g__Akkermansia | ABX-H-QSQSF | 4.03 | 0.01 |
| g__Bilophila | ABX-H-QSQSF | 3 | 0.02 |
| g__Anaerostipes | ABX-H-QSQSF | 2.86 | 0.01 |
| g__unclassified_f__Enterobacteriaceae | ABX-H-QSQSF | 2.54 | 0.02 |
| g__norank_f__Muribaculaceae | ABX-H-QSQSF | 4.99 | 0.03 |
| g__norank_o__Rhodospirillales | ABX-H-QSQSF | 3.31 | 0 |
| g__Parabacteroides | ABX-H-QSQSF | 4.11 | 0.01 |
| g__Anaerofustis | ABX-H-QSQSF | 2.62 | 0.04 |
| g__Flavonifractor | ABX-H-QSQSF | 2.65 | 0 |
| g__Turicimonas | ABX-H-QSQSF | 3.26 | 0 |
| g__norank_f__Eggerthellaceae | ABX-H-QSQSF | 2.05 | 0.04 |

Abbreviations: QTQSF, Qitu Qushi Formula; ABX, antibiotics; LDA, linear discriminant analysis.
