# Supplementary material for: Qitu qushi formula ameliorates diabetic kidney disease potentially through gut microbiota-derived indole-3-propionic Acid–Mediated regulation of the Sirt1/FoxO1 pathway
Source: Front Pharmacol. 2026 Jun 2;17:1802567. doi: 10.3389/fphar.2026.1802567 (PMC13269076; doi:10.3389/fphar.2026.1802567)
Supplement: Supplementary file 9 [file Table3.docx]

Table S3 Differential metabolites in patients with diabetic kidney disease before and after treatment (VIP>1, P<0.05).

| Metabolites | P value | VIP Score | Post-treatment Trend |
| --- | --- | --- | --- |
| 9-Oxo-10-acridineacetic acid | 0.01 | 4.88 | ↑ |
| N-Acetyltyrosine | 0 | 4.55 | ↑ |
| Gemfibrozil | 0 | 4.25 | ↑ |
| Vomifoliol | 0 | 4.23 | ↑ |
| 4-(Methylthio)-1-butanol | 0.02 | 4.13 | ↑ |
| 22-Angeloylbarringtogenol C | 0.02 | 4.01 | ↑ |
| Equilenin | 0.04 | 3.8 | ↑ |
| (+)-Lysergic acid | 0.03 | 3.3 | ↑ |
| 6-Thiourate | 0 | 3 | ↑ |
| Germacrone 4,5-epoxide | 0 | 2.94 | ↑ |
| 3a,17a-Dihydroxy-5b-androstane | 0.05 | 2.89 | ↑ |
| 4-Hydroxy-3-methoxy-cinnamoylglycine | 0.03 | 2.81 | ↑ |
| 7-Amino-4-methylcoumarin | 0 | 2.7 | ↑ |
| Sphingosine 1-phosphate | 0.03 | 2.65 | ↑ |
| 11-Hydroxyyohimbine | 0.04 | 2.65 | ↑ |
| Piperic acid | 0.01 | 2.63 | ↑ |
| DG(i-22:0/PGE1/0:0) | 0.02 | 2.63 | ↑ |
| 4-amino-4-deoxychorismate | 0.01 | 2.59 | ↑ |
| Varenicline Tartrate | 0.05 | 2.54 | ↑ |
| Phenol sulphate | 0 | 2.53 | ↑ |
| Hesperetin | 0.01 | 2.5 | ↑ |
| 5,8,11-Eicosatrienoic acid | 0.03 | 2.35 | ↑ |
| Ferreirin | 0.01 | 2.34 | ↑ |
| (-)-Nopol | 0.01 | 2.3 | ↑ |
| DG(2:0/LTE4/0:0) | 0.03 | 2.3 | ↑ |
| 10-Hydroxy-3-methoxy-1,3,5,7-cadinatetraen-9-one | 0.02 | 2.29 | ↑ |
| Betavulgarin | 0 | 2.21 | ↑ |
| 3-Dehydroteasterone | 0.02 | 2.15 | ↑ |
| Meproscillarin | 0.05 | 2.1 | ↑ |
| Penitrem D | 0.03 | 2.01 | ↑ |
| Apigenin | 0.01 | 2 | ↑ |
| Eugenin | 0.04 | 2 | ↑ |
| Capsiate | 0.01 | 1.89 | ↑ |
| Adb-chminaca, (+/-)- | 0.04 | 1.88 | ↑ |
| 2-Tiglylcarnitine | 0.05 | 1.86 | ↑ |
| Coumarin | 0 | 1.85 | ↑ |
| 3-Oxo-2-(2-entenyl)cyclopentaneoctanoic acid | 0.01 | 1.83 | ↑ |
| Pyrroline | 0 | 1.76 | ↑ |
| Sarmentosin | 0.03 | 1.61 | ↑ |
| Arctiopicrin | 0.02 | 1.6 | ↑ |
| 2,4-Dichlorophenylacetic acid | 0 | 1.6 | ↑ |
| 5-Methyl-2-furaldehyde | 0 | 1.51 | ↑ |
| Alpha-Irone | 0.02 | 1.51 | ↑ |
| Tyramine glucuronide | 0.04 | 1.5 | ↑ |
| 2,3-Dihydroxycarbamazepine | 0.04 | 1.49 | ↑ |
| Phenethyl rutinoside | 0.01 | 1.49 | ↑ |
| 2-(2-Aminopropanoylamino)bicyclo[3.1.0]hexane-2,6-dicarboxylic acid | 0.04 | 1.47 | ↑ |
| Pubesenolide | 0.05 | 1.42 | ↑ |
| 2-Hydroxyphenylacetic Acid | 0.02 | 1.41 | ↑ |
| Kinetensin 1-3 | 0.01 | 1.4 | ↑ |
| DUDP | 0.04 | 1.4 | ↑ |
| PA(18:1(11Z)/22:6(4Z,7Z,10Z,13Z,16Z,19Z)) | 0.01 | 1.34 | ↑ |
| (Z)-4-Hydroxy-6-dodecenoic acid lactone | 0 | 1.27 | ↑ |
| Cannabigerolate | 0.04 | 1.21 | ↑ |
| Alpha-curcumene | 0.01 | 1.2 | ↑ |
| Indole-3-acetaldehyde | 0 | 1.13 | ↑ |
| Carnosic acid | 0.03 | 1.12 | ↑ |
| Methyl Cinnamate | 0.01 | 1.12 | ↑ |
| Oxindole | 0 | 1.1 | ↑ |
| (+)-Neomenthyl acetate | 0.02 | 1.05 | ↑ |
| 4-Heptylphenol | 0 | 1.02 | ↑ |
| Linoleyl-l-carnitine | 0 | 3.73 | ↓ |
| Cochliobolin A | 0.01 | 3.61 | ↓ |
| N-Stearoyl Isoleucine | 0.01 | 3.57 | ↓ |
| N-Stearoyl Cysteine | 0.03 | 3.44 | ↓ |
| Tryptophyl-Threonine | 0.01 | 3.27 | ↓ |
| Tryptophyl-Proline | 0.01 | 3.14 | ↓ |
| Acolongifloriside K | 0.04 | 3.05 | ↓ |
| Uric Acid | 0 | 2.87 | ↓ |
| Tropolone | 0.04 | 2.83 | ↓ |
| (1S,2R)-1-C-(indol-3-yl)glycerol 3-phosphate | 0.04 | 2.57 | ↓ |
| 5-Methoxytryptophan | 0.02 | 2.32 | ↓ |
| L-prolinamide | 0 | 2.27 | ↓ |
| 4,4-Dimethyl-2-[3-carboxylatopropyl]-2-tridecyloxazolidine 3-oxide | 0.04 | 2.25 | ↓ |
| Chenodeoxycholylalanine | 0 | 2.22 | ↓ |
| 3,4-Dihydro-2H-1-benzopyran-2-one | 0.01 | 2.07 | ↓ |
| Aceneuramic acid | 0.03 | 2.07 | ↓ |
| Peiminine | 0.02 | 1.94 | ↓ |
| Nonanedioylcarnitine | 0.04 | 1.9 | ↓ |
| Isolithocholic acid | 0 | 1.89 | ↓ |
| Apo-14'-zeaxanthinal | 0.04 | 1.89 | ↓ |
| N-Stearoyl Valine | 0.03 | 1.88 | ↓ |
| Amrinone | 0.03 | 1.76 | ↓ |
| Delphinidin 3-glucoside | 0.04 | 1.72 | ↓ |
| Normorphine | 0.02 | 1.67 | ↓ |
| 3-Indoleacetic Acid | 0.04 | 1.66 | ↓ |
| THIARABINE | 0.01 | 1.63 | ↓ |
| Heptadecadienoic acid | 0.02 | 1.54 | ↓ |
| 5-Methoxytryptophol | 0.03 | 1.52 | ↓ |
| 2-((3,5-Dihydroxyphenyl)amino)acetic acid | 0.01 | 1.51 | ↓ |
| Isoleucylhydroxyproline | 0.05 | 1.47 | ↓ |
| Tryptophyl-Aspartate | 0.01 | 1.47 | ↓ |
| 3-[[5-Methyl-2-(1-methylethyl)cyclohexyl]oxy]-1,2-propanediol | 0.05 | 1.44 | ↓ |
| L-Formylkynurenine | 0.02 | 1.42 | ↓ |
| N-Docosahexaenoyl Leucine | 0.01 | 1.33 | ↓ |
| Fangchinoline | 0.04 | 1.23 | ↓ |
| N-Acetyl-D-phenylalanine | 0.01 | 1.22 | ↓ |
| Oryzalide B | 0.03 | 1.19 | ↓ |
| N-Palmitoyl Isoleucine | 0.02 | 1.19 | ↓ |
| Aceclofenac | 0.01 | 1.18 | ↓ |
| 4-Chloro-L-phenylalanine | 0 | 1.14 | ↓ |
| 11-Hydroxyheptadecanoylcarnitine | 0 | 1.12 | ↓ |
| L-Phenylalanine | 0 | 1.04 | ↓ |

Abbreviations: VIP, Variable Importance in Projection.
