# Supplementary material for: Qitu qushi formula ameliorates diabetic kidney disease potentially through gut microbiota-derived indole-3-propionic Acid–Mediated regulation of the Sirt1/FoxO1 pathway
Source: Front Pharmacol. 2026 Jun 2;17:1802567. doi: 10.3389/fphar.2026.1802567 (PMC13269076; doi:10.3389/fphar.2026.1802567)
Supplement: Supplementary file 10 [file Table4.docx]

Table S4 KEGG pathway enrichment of differential metabolites between before and after treatment in patients with DKD.

| Pathway Name | Metabolites enriched in the pathway and their post-treatment trend | P value | Adjusted P value | Impact value |
| --- | --- | --- | --- | --- |
| Benzoxazinoid biosynthesis | Oxindole↑, (1S,2R)-1-C-(indol-3-yl)glycerol 3-phosphate/(1S,2R)-1-C-(indol-3-yl)↓ | 0.001 | 0.011 | 0.143 |
| Tryptophan metabolism | Indole-3-acetaldehyde↑, 3-Indoleacetic Acid↓, L-Formylkynurenin↓ | 0.002 | 0.015 | 0.130 |
| Phenylalanine metabolism | L-Phenylalanine↓, 2-Hydroxyphenylacetic Acid↑, N-Acetyl-D-phenylalanine↓ | 0.001 | 0.011 | 0.087 |
| Phenylalanine, tyrosine and tryptophan biosynthesis | L-Phenylalanine↓, (1S,2R)-1-C-(indol-3-yl)glycerol 3-phosphate/(1S,2R)-1-C-(indol-3-yl)↓ | 0.010 | 0.058 | 0.025 |

Abbreviations: KEGG, Kyoto Encyclopedia of Genes and Genomes; DKD, diabetic kidney disease.
