# Supplementary material for: Qitu qushi formula ameliorates diabetic kidney disease potentially through gut microbiota-derived indole-3-propionic Acid–Mediated regulation of the Sirt1/FoxO1 pathway
Source: Front Pharmacol. 2026 Jun 2;17:1802567. doi: 10.3389/fphar.2026.1802567 (PMC13269076; doi:10.3389/fphar.2026.1802567)
Supplement: Supplementary file 11 [file Table6.docx]

Table S6 Differentially abundant gut microbial taxa in mice following fecal microbiota transplantation (LDA>2, P<0.05).

| Differential Taxa | Predominant group | LDA Score |
| --- | --- | --- |
| g__Hydrogenoanaerobacterium | H-QTQSF+FMT | 2.11 |
| g__Monoglobus | H-QTQSF+FMT | 3.08 |
| g__Negativibacillus | H-QTQSF+FMT | 2.36 |
| g__Ruminiclostridium | H-QTQSF+FMT | 2.42 |
| g__Family_XIII_AD3011_group | H-QTQSF+FMT | 2.88 |
| g__Bifidobacterium | H-QTQSF+FMT | 2.33 |
| g__Longibaculum | H-QTQSF+FMT | 2.38 |
| g__Christensenellaceae_R-7_group | H-QTQSF+FMT | 2.23 |
| g__unclassified_p__Bacillota | H-QTQSF+FMT | 2.34 |
| g__Mucispirillum | H-QTQSF+FMT | 2.64 |
| g__unclassified_f__Eggerthellaceae | H-QTQSF+FMT | 2.23 |
| g__Rikenella | H-QTQSF+FMT | 4.13 |
| g__Rikenellaceae_RC9_gut_group | H-QTQSF+FMT | 3.89 |
| g__Harryflintia | H-QTQSF+FMT | 2.37 |
| g__Anaerotignum | H-QTQSF+FMT | 2.23 |
| g__Butyricimonas | H-QTQSF+FMT | 2.13 |
| g__unclassified_c__Clostridia | H-QTQSF+FMT | 2.3 |
| g__NK4A214_group | H-QTQSF+FMT | 2.85 |
| g__Candidatus_Arthromitus | H-QTQSF+FMT | 2.67 |
| g__unclassified_f__Ruminococcaceae | H-QTQSF+FMT | 3.13 |
| g__Anaerotruncus | H-QTQSF+FMT | 3.21 |
| g__norank_o__Oscillospirales | ABX-H-QTQSF+FMT | 2.94 |
| g__norank_f__Gastranaerophilaceae | ABX-H-QTQSF+FMT | 2.62 |
| g__norank_f__UCG-010 | ABX-H-QTQSF+FMT | 2.73 |
| g__[Eubacterium]_xylanophilum_group | ABX-H-QTQSF+FMT | 3.66 |
| g__UCG-003 | ABX-H-QTQSF+FMT | 2.42 |
| g__Odoribacter | ABX-H-QTQSF+FMT | 3.74 |
| g__norank_f__Flavobacteriaceae | ABX-H-QTQSF+FMT | 2.84 |
| g__Prevotellaceae_NK3B31_group | ABX-H-QTQSF+FMT | 4.31 |
| g__norank_o__Clostridia_UCG-014 | ABX-H-QTQSF+FMT | 3.58 |
| g__Family_XIII_UCG-001 | ABX-H-QTQSF+FMT | 2.47 |
| g__Lachnospiraceae_FCS020_group | ABX-H-QTQSF+FMT | 2.1 |
| g__norank_o__RF39 | ABX-H-QTQSF+FMT | 2.21 |
| g__Turicibacter | ABX-H-QTQSF+FMT | 2.51 |
| g__Alistipes | ABX-H-QTQSF+FMT | 4.26 |
| g__unclassified_f__Prevotellaceae | ABX-H-QTQSF+FMT | 2.73 |
| g__Tyzzerella | ABX-H-QTQSF+FMT | 2.31 |
| g__Ruminococcus | ABX-H-QTQSF+FMT | 3.57 |
| g__Gordonibacter | ABX-H-QTQSF+FMT | 2.6 |
| g__norank_f__Rs-E47_termite_group | ABX-H-QTQSF+FMT | 3.22 |
| g__Anaerofustis | ABX-H-QTQSF+FMT | 2.21 |
| g__norank_c__Clostridia | ABX-H-QTQSF+FMT | 2.44 |
| g__norank_f__Anaerovoracaceae | ABX-H-QTQSF+FMT | 2.86 |
| g__GCA-900066575 | ABX-H-QTQSF+FMT | 2.96 |
| g__Zag_111 | ABX-H-QTQSF+FMT | 2.2 |
| g__Acetatifactor | ABX-H-QTQSF+FMT | 2.11 |
| g__Candidatus_Saccharimonas | ABX-H-QTQSF+FMT | 3.53 |
| g__[Eubacterium]_siraeum_group | ABX-H-QTQSF+FMT | 3.87 |
| g__[Clostridium]_innocuum_group | ABX | 2.45 |
| g__Anaerostipes | ABX | 2.98 |
| g__unclassified_f__Enterobacteriaceae | ABX | 2.48 |
| g__unclassified_f__Sutterellaceae | ABX | 2.49 |
| g__norank_f__Muribaculaceae | ABX | 4.93 |
| g__norank_o__Rhodospirillales | ABX | 2.8 |

Abbreviations: QTQSF, Qitu Qushi Formula; FMT, fecal microbiota transplantation; ABX, antibiotics; LDA, linear discriminant analysis.
